# Properties of human genes guided by their enrichment in rare and common variants

**DOI:** 10.1002/humu.23377

**Published:** 2017-12-21

**Authors:** Eman Alhuzimi, Luis G. Leal, Michael J.E. Sternberg, Alessia David

**Affiliations:** ^1^ Structural Bioinformatics Group, Department of Life Sciences Imperial College London London SW7 2AZ UK

**Keywords:** genetic variants, human disease, protein coding genes, protein network

## Abstract

We analyzed 563,099 common (minor allele frequency, MAF≥0.01) and rare (MAF < 0.01) genetic variants annotated in ExAC and UniProt and 26,884 disease‐causing variants from ClinVar and UniProt occurring in the coding region of 17,975 human protein‐coding genes. Three novel sets of genes were identified: those enriched in rare variants (*n* = 32 genes), in common variants (*n* = 282 genes), and in disease‐causing variants (*n* = 800 genes). Genes enriched in rare variants have far greater similarities in terms of biological and network properties to genes enriched in disease‐causing variants, than to genes enriched in common variants. However, in half of the genes enriched in rare variants (*AOC2*, *MAMDC4*, *ANKHD1*, *CDC42BPB*, *SPAG5*, *TRRAP*, *TANC2*, *IQCH*, *USP54*, *SRRM2*, *DOPEY2*, and *PITPNM1*), no disease‐causing variants have been identified in major, publicly available databases. Thus, genetic variants in these genes are strong candidates for disease and their identification, as part of sequencing studies, should prompt further *in vitro* analyses.

The achievement of personalized medicine, which is the prevention and treatment of human disease by taking individual genetic variability into account, is one of the main goals of modern medicine. Nevertheless, the interpretation of the large amount of genetic data that next‐generation sequencing technology is delivering remains one of the major challenges preventing us from achieving this goal. This is especially true for rare variants, which occur at a low frequency in the population and account for a large proportion of genetic variations identified in an individual's genome. Rare variants are likely to being involved in the pathogenesis of oligogenic disorders, as well as represent the missing heritability of common conditions, such as diabetes and cancer. Indeed, one is compelled to ask whether specific genes are enriched in rare variants, similar to what is observed in disease‐causing variants. And if so, what are the characteristics of genes enriched in rare variants? These important questions remain unanswered.

The recent availability of large, publicly available databases of genetic variations provides us with the unprecedented opportunity to analyze the distribution of rare and common variants across our genome. In this study, we combined information from the ExAC database (Lek et al., 2016), with data from dbSNP (Sherry et al., 2001), ClinVar (Landrum et al., 2016), and UniProt (UniProt Consortium, 2015), to explore the distribution of rare and common variants in protein‐coding genes and to compare the characteristics of genes enriched in rare or common variants with those of genes enriched in disease‐causing variants. As the focus of our analysis was on short variants occurring in the exome, we made the arbitrary decision to not include variants annotated as “downstream gene,” “3′ UTR,” and “5′ UTR” in the analysis.

We analyzed 563,099 genetic variants with no disease association (481,277 rare variants minor allele frequency [MAF] < 0.01 and 81,822 common variants MAF ≥ 0.01) and 26,884 disease‐causing variants distributed across 17,975 protein‐coding genes (the construction of the dataset and the list of genes enriched in variants is presented in the Supplementary Material). For variants not reported as disease‐causing, we required a global MAF, which was retrieved from ExAC or dbSNP (if not present in ExAC). These variants were further classified according to their reported global MAF in rare (MAF < 0.01) and common (MAF ≥ 0.01). Disease‐causing variants were distributed across 2,631 protein‐coding genes, whereas rare and common variants were distributed across 17,540 and 15,391 genes, respectively. The hypergeometric test was used to assess whether rare, common, or disease‐causing variants occurred more often than expected in certain genes (gene enrichment). The Benjamini–Hochberg correction (Benjamini & Hochberg, 1995) was applied to adjust for multiple comparisons (see Supplementary Material). We found that 800 genes were enriched in disease‐causing variants (disease‐EVset), 32 genes in rare variants (rare‐EVset), and 282 genes in common variants (common‐EVset), with no overlap between the three sets. Nevertheless, since the identification of the three sets was based on enrichment of specific types of variants (rare, common, or disease‐causing), disease‐causing variants could still be present in genes included in the common‐EVset and rare‐EVset.

Genes involved in the pathogenesis of disease have been shown to be under strong or moderate purifying selection (Collins, 2015; Quintana‐Murci, 2016). As the evidence of a gene's enrichment in rare variants should reflect its selection constrains, we expected these 32 novel genes enriched in rare variants to be under selective pressure. Indeed, all but three genes in the rare‐EVset were predicted to be under moderate purifying selection when assessed using the McDonald–Kreitman neutrality index implemented in the Gene Damage Index (GDI) server (Itan et al., 2015).

We used the pLi scores and dN/dS ratio (see Supplementary Material) to characterize and compare genes in the three novel enriched sets. Genes associated with disease have been shown to have a high pLi score and a low dN/dS ratio, which indicate that they are under selective pressure (Ge, Kwok, & Shieh, 2015; Lek et al., 2016). In particular, loss‐of‐function (LoF) tolerant genes have been shown to have a pLi score ≤ 0.1, whereas highly constrained genes a pLi ≥ 0.9. We found that genes in the disease‐EVset and rare‐EVset had a significantly higher pLi score (*P* < 0.0001 Kruskal–Wallis Rank Sum test; Supp. Table S1) and lower dN/dS ratio (*P* < 0.0001) compared with genes in the common‐EVset. However, there was no difference between genes in the disease‐EVset versus rare‐EVset (*P* = 0.21 for pLi scores and *P* = 0.52 for dN/dS ratio, Mann–Whitney test, two tailed; Supp. Figure S1). Interestingly, 13 out of 32 genes from the rare‐EVset (*TRRAP*, *WDFY3*, *KMT2C*, *HECTD4*, *CHD7*, *ANKHD1*, *CDC42BPB*, *SRCAP*, *NOTCH1, BSN*, *TANC2*, *CELSR3*, and *PITPNM1*) had a pLi score ≥ 0.9, which is similar to the scores identified for haploinsufficient genes associated with the most severe and early onset phenotypes (Lek et al., 2016).

Genes involved in disease have well‐established properties, such as enrichment for essential genes (Dickinson et al., 2016) and centrality in the protein interactome (Barrenas, Chavali, Holme, Mobini, & Benson, 2009; Barabási, Gulbahce, & Loscalzo, 2011), which distinguish them from nondisease‐causing genes. The rare‐EVset and common‐EVset, however, are novel sets of genes. We assessed whether these two sets differ in their biological properties, and whether genes in the rare‐EVset share similar biological and network properties with genes in the disease‐EVset, as this could indicate that genes in the rare‐EVset also harbor deleterious genetic variants.

We first explored the disease‐EVset, rare‐EVset, and common‐EVset for enrichment in essential genes (Figure 1 and Supp. Tables S2A–S2D). Genes were classified as essential when the mouse ortholog was classified as essential, or if the gene was reported in the Online GEne Essentiality (OGEE) database (Chen, Minguez, Lercher, & Bork, 2012) (see Supplementary Material). Although we found no significant difference in the frequency of gene essentiality between the disease‐EVset (511 essential genes, 64%) and the rare‐EVset (17 genes, 53%), essential genes were more likely to be present in the rare‐EVset compared with the common‐EVset (30 genes, 11%, *P* < 0.001). Similar results were obtained when genes in the three sets were analyzed in terms of pathways and Gene Ontology (GO) terms, as detailed in Supplementary Material.

**Figure 1:**
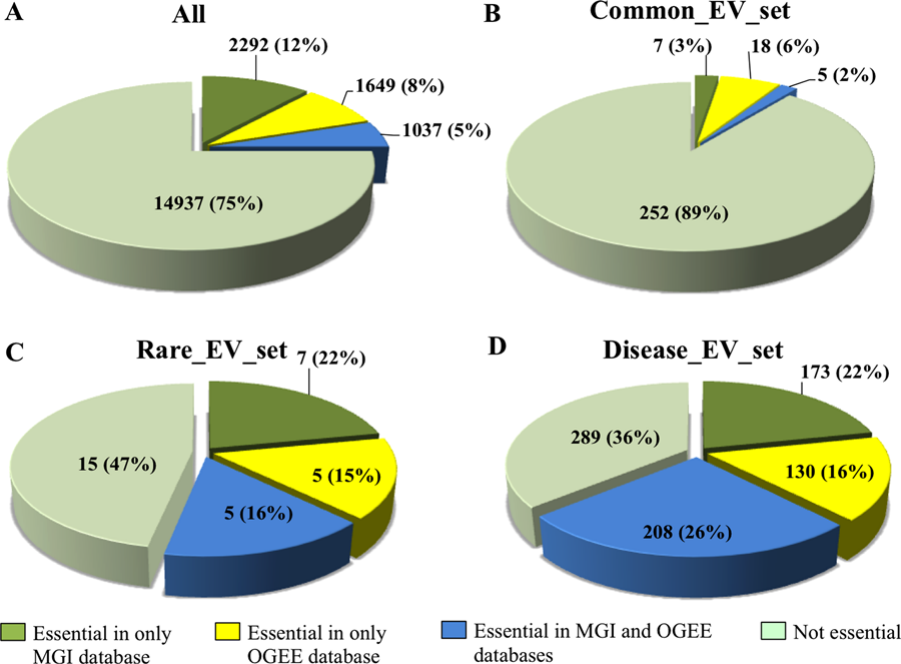
Essential genes. Number (percentage) of essential genes in: (**A**) the set of all human protein‐coding genes reported in UniProt, (**B**) genes enriched in common variants (common‐EVset), (**C**) genes enriched in rare variants (rare‐EVset), and (**D**) genes enriched in disease‐causing variants (disease‐EVset). Genes are classified as essential if the mouse ortholog of the human gene produces a lethal phenotype (essential in MGI database) or if the gene is reported in the OGEE database (essential in OGEE)

We examined the network properties of genes in the rare‐EV and common‐EV sets and compared them with those of genes in the disease‐EVset, by mapping 279,904 non‐redundant protein‐protein interaction data for 21,274 human genes extracted from BioGRID (Chatr‐Aryamontri et al., 2015). Interestingly, only 57% of the genes in the common‐EVset had at least one known interactor, compared to 93% of genes in the disease‐EVset and 97% of genes in the rare‐EVset (*P* < 0.001 for disease‐EVset vs. common‐EVset and *P* < 0.001 for rare‐EVset vs. common‐EVset). Genes in the disease‐EVset and rare‐EVset were equally likely to participate in several interactions (disease‐EVset: median number of interactors 14, range 0–2,064; rare‐EVset: median number of interactors 21.5, range 0–234; *P* value 0.18). Moreover, genes in the common‐EVset were less likely to participate in several interactions, compared with genes in the disease‐EVset or rare‐EVset (common‐EVset number of interactors: median 1, range 0–317; Benjamini–Hochberg adjusted *P* value < 0.05 for rare‐EVset vs. common‐EVset and for disease‐EVset vs. common‐EVset). Surprisingly, when we examined the first‐degree neighbors (direct interaction partners), we found that the three sets of enriched genes were part of a highly connected network. Out of a total of 1,115 enriched genes, 682 (565 in the disease‐EVset, 23 in the rare‐EVset, and 94 in the common‐EVset) were adjacent nodes in the gene network comprising of a 682 nodes and 1863 edges. A greater number of genes from the rare‐EVset (23 of 32) rather than common‐EVset (94 of 282) were part of this highly connected network (*P* < 0.001). This further supports our finding that genes enriched in rare variants (rare‐EVset) are biologically different compared to genes enriched in common variants (common‐EVset) and similar to genes enriched in disease‐causing variants, suggesting that the vast majority of genes from the rare‐EVset could also be involved in the pathogenesis of disease.

One of the limitations of our study is that we only included in our analysis short variants occurring in the exome and did not include variants, such as those occurring in 3′UTR and 5′ UTR. Moreover, compared with the pLi score, which identified over 3000 LoF intolerant genes, our strategy appears to have a lower discovery rate, which may reflect a lower sensitivity. Furthermore, we acknowledge that some databases used for our analyses, such as the protein interactions and pathways databases, may be biased toward proteins that, because of their involvement in disease, have been studied more extensively. Nevertheless, the consistency of our results, which also include data from less biased sources, such as GO, OGEE, and the mouse database, strongly supports the notion that genes enriched in rare‐variants share similar biological properties with genes enriched in disease‐causing variants. We therefore expected all, or the majority, of the 32 genes enriched in rare‐variants to be annotated as causing disease. Surprisingly, only 16 (50%) of these genes were reported as involved in the pathogenesis of disease in the three major databases of genetic variants (ClinVar, OMIM, and UniProt). Indeed, the suggestion that the rare‐EVset may have several disease‐associated genes is consistent with the fact that the rare‐EVset had significantly more genes with genetic variants known to cause disease compared to the common‐EVset (*P* < 0.001). We examined the GWAS Catalog (release November 2016), which reports associations for 19,849 genes and 1,591 phenotypes. We found that an additional four genes (*HECTD4*, *BSN*, *WDR6*, and *SGSM3*) enriched in rare variants were annotated as significantly associated with disease (GWAS *P *≤ 5 × 10^−8^). When we examined the common‐EVset, an additional 56 out of 282 genes were reported associated with disease, which confirmed that the rare‐EVset is enriched in genes causing or associated with disease compared with the common‐EVset: 20 (62.5%) genes in total causing or with an association to disease in the rare‐EVset versus 91 (32.3%) genes in the common‐EVset, *P* < 0.001 (Supp. Table S3).

In the 12 remaining genes in the rare‐EVset, no known involvement in disease was found, when the presence of large deletions and duplications (>50 kb) was excluded, by interrogating ClinVar, UniProt, OMIM, and GWAS catalog. To further explore the presence of pathogenic variants in these genes, we also interrogated the HGMD database (Supp. Table S4). Four genes (*AOC2*, *ANKHD1*, *IQCH*, and *DOPEY2*) were not found in the HGMD database; four genes (*CDC42BPB*, *TRRAP*, *TANC2*, and *USP54*) had variants of uncertain clinical significance annotated; the remaining four genes (*MAMDC4*, *SPAG5*, *SRRM2*, and *PITPNM1*) were present in HGMD, but no variants data were reported in the publicly available version. Since this version does not include the most recently deposited entries, we interrogated the DECIPHER database for known pathogenic genetic variants. DECIPHER is used by the clinical and genetics community and clinician scientists involved in the 100K Genomes Project to share phenotypic and genotypic data. It contains up‐to‐date, highly curated information. Although copy number gain and loss were reported in these genes, no variants were annotated, with the exception of one frameshift variant of “uncertain” clinical significance in *SRRM2*.

These 12 genes are compelling candidates for harboring short variants causing or increasing the risk of disease. LoF score, which indicates the tolerance of a gene to nonsense, splice acceptor, and splice donor variants caused by single nucleotide changes (Lek et al., 2016) was available in the ExAC database for 11 out 12 genes. Four of the 12 genes (*ANKHD1*, *CDC42BPB*, *TRRAP*, and *TANC2*) were reported as extremely LoF intolerant (pLi score = 1.00; Supp. Table S5). Indeed, with the exclusion of USP54, which had no amino acid substitutions, a large percentage of missense variants in each gene was predicted damaging by SIFT (Kumar, Henikoff, & Ng, 2009) (median: 48.8%, range 38.8%–73.4%), PolyPhen‐2 (Adzhubei et al., 2010) (median: 49.1%, range 33.3%–57.5%), and MSC‐corrected CADD scores (Itan et al., 2016) (median: 51.6% range 11.3%–59.4%; Supp. Table S6). Moreover, the median CADD C‐scores (Kircher et al., 2014) for the 12 genes ranged from 13.9 to 23.4 for missense variants and from 36.1 to 45.0 for nonsense variants (Supp. Figure S2). Of notice, the median C‐scores for nonsense variants in our 12 genes enriched in rare variants were similar to the median values observed in genes harboring disease‐causing variants or variants associated with complex traits (Kircher et al., 2014).

In order to describe these 12 genes at population level, we extracted population allele frequencies from the Genome Aggregation Database (gnomAD). We observed that 27%–45% of variants for each gene (median 40%) were not population specific (Supp. Figure S3). All common variants (MAF ≥ 0.01) were not population specific, as they were observed in at least two populations. Overall, a higher proportion of common variants was observed for all genes in the Ashkenazi Jewish and Finnish populations compared to the other populations (Supp. Figure S4). When investigating the differences in allele frequencies among different populations, we observed a change from rare to common in 278 variants across different populations. Of note, one start loss (rs145549199) and 17 missense variants (rs61758138, rs536168385, rs35833794, rs34351794, rs34625494, rs117132686, rs142091518, rs143714922, rs186097368, rs140559332, rs202115673, rs376290390, rs143024358, rs117133016, rs114899013, rs138495768, and rs114848780) located in *ANKHD1*, *AOC2*, *DOPEY2*, *MAMDC4*, *SPAG5*, and *SRRM2* are predicted damaging by SIFT, PolyPhen‐2, and CADD (Supp. Table S7). These variants could be important candidates when exploring causes for differences in disease predisposition in different populations.

In order to explore whether these 12 genes enriched in rare variants are closely related to each other, we looked for enrichment in pathways, GO terms or protein domains. However, no enrichment was found. Moreover, when the human gene connectome (Itan et al., 2013) was applied, these genes were not in close proximity (median small biological distance 16.4, range 10–44; Supp. Table S8).

Most of the 12 novel genes enriched in rare variants were predicted “intolerant” to genetic variations by several gene‐level metrics (Supp. Table S9). In particular, the RVIS matrix (Petrovski, Wang, Heinzen, Allen, & Goldstein, 2013) showed that, with the exception of *USP54* and *MAMDC4*, all other genes had a negative RVIS score suggestive of genic intolerance. Moreover, over one half of these genes (*ANKHD1*, *CDC42BPB*, *PITPNM1*, *SRRM2*, *TRRAP*, *TANC2*, and *DOPEY2*) were in the top 10% percentile for the most intolerant human genes. Similar results were obtained when using the “functional indispensability” score (Khurana, Fu, Chen, & Gerstein, 2013), which is calculated based on a gene's functional and evolutionary properties. High functional indispensability scores were indeed present for *ANKHD1*, *CDC42BPB*, *PITPNM1*, *SRRM2*, *TRRAP*, and *AOC2*. Furthermore, *TRRAP* and *PITPNM1* were among the top excessively constrained genes of the human genome, when assessed using the DNE gene‐level method (Samocha et al., 2014). Interestingly, when the GDI scores were used, all 12 genes showed a “medium” GDI prediction score, thus placing them in‐between the set of genes associated with embryo‐lethal disorders (low GDI score) and the set of genes that are unlikely to cause monogenic disorders (high GDI score) (Itan et al., 2015). Moreover, with the exception of *MAMDC4* and *ANKHD1*, all genes were predicted to be under moderate purifying selection when assessed using the McDonald–Kreitman neutrality index implemented in the GDI server (Itan et al., 2015).

Among the 12 genes within the rare‐EVset, *CDC42BPB*, *DOPEY2*, and *IQCH* are reported in the GWAS Catalog with a *P* value between 5 × 10^−6^ and 5 × 10^−8^ and are associated with B cell lymphoma, schizophrenia, and age on onset of menarche, respectively. Next, we interrogated the DisGeNET database (Piñero et al., 2017), which also includes disease associations predicted using mouse and rat genome databases and text‐mining‐derived associations with Mendelian, complex, and environmental diseases. An association with disease was reported for 10 out of 12 genes. Among these, *SRRM2* was a candidate gene for amyotrophic lateral sclerosis, *AOC2* for diabetes mellitus and *PITPNM1* for schizophrenia. When we examined the first‐degree neighbors of these 12 genes enriched in rare variants but with no known disease‐causing genetic variants, several first‐degree interactors of *SRRM2*, *SPAG5*, *AOC2*, and *TRRAP* were disease causing (Figure 2). This makes these four genes strong candidates for harboring disease‐causing genetic variants, based on the widely accepted guilty‐to‐association principle. Indeed, a role for *TRRAP* as an oncogene has been proposed, with a recurrent somatic mutation (p.Ser722Phe) identified in six out of 167 patients with melanoma (Wei et al., 2011). The pLi score for *TRRAP* is 1.00 (pLi scores for all 12 genes are reported in Supp. Table S5). Moreover, our analysis of first‐degree neighbors suggests that *TRRAP*, *SRRM2*, and *SPAG5* could be pleiotropic genes involved in the pathogenesis of several disorders. Within the protein network, all these genes have first‐degree neighbors involved in neoplasms, congenital, and neurological disorders. These classes of diseases have all been shown to be enriched in disease‐causing pleiotropic genes (Ittisoponpisan, Alhuzimi, Sternberg, & David, 2017).

**Figure 2:**
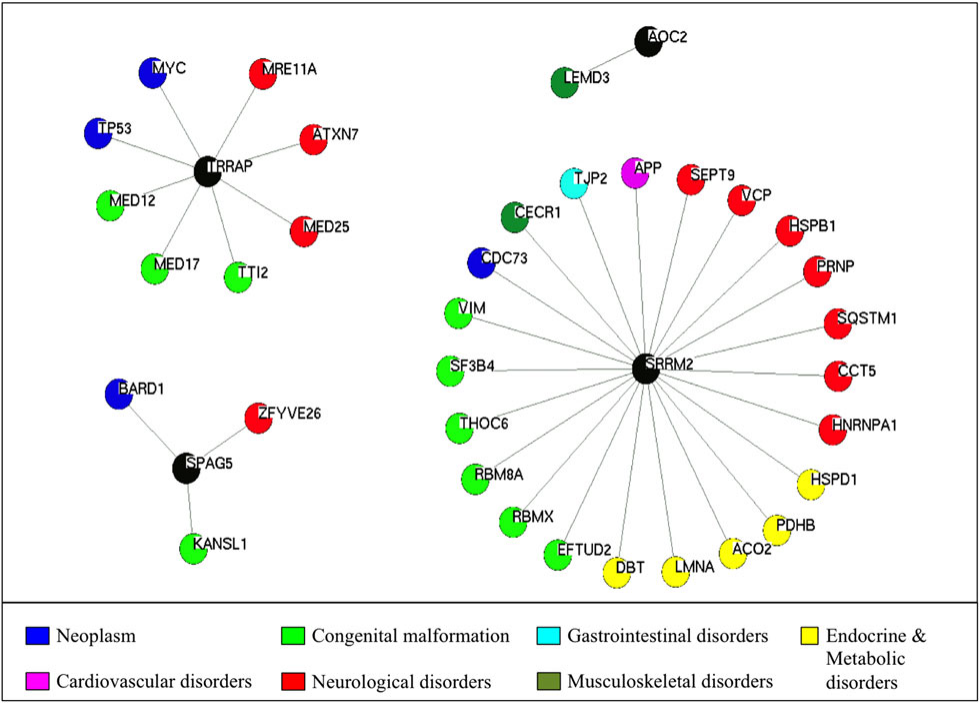
First‐degree neighbors for *TRRAP*, *AOC2*, *SRRM2*, and *SPAG5*. Only first‐degree neighbors with known disease‐causing variants are displayed. *TRRAP*, *AOC2*, *SRRM2*, and *SPAG5* are presented as black circles. Diseases are classified according to the 10th revision of the International Statistical Classification of Diseases and Related Health Problems (ICD‐10)

Rare variants are likely contributors to the phenotypic variations observed in the population and to the increased risk of disease and may represent the missing heritability. However, GWAS studies still lack the statistical power to identify rare variants, and imputation reference panels fail to tag them (Bomba, Walter, & Soranzo, 2017). Other methods, such as the burden test and targeted‐region sequencing are, thus, often used to identify associations between rare variants and disease (Lee, Abecasis, Boehnke, & Lin, 2014). We identified a novel set of genes, which have biological properties similar to those of disease‐causing genes and are enriched in rare variants. Such knowledge could be added to currently available tests and algorithms to boost their power to detect disease associations. It has, indeed, been shown that the power to detect association increases when only variants predicted deleterious are used (Bomba, Walter, & Soranzo, 2017). Similarly, taking into account the biological properties of genes harboring rare variants could help boost the power to detect meaningful associations, as well as aid in the interpretation of the results of on‐going and future sequencing studies.

In conclusion, we identified two novel sets of genes, enriched in either rare or common variants. We showed that genes in the rare‐EVset are biologically different to genes in the common‐EVset and share biological and network properties with genes enriched in disease‐causing variants. To date, only half of the genes in the rare‐EVset have genetic variants associated with human disease. Nevertheless, the remaining genes from the rare‐EVset are also strong candidates for disease, as suggested by the concordant results obtained from several well‐established tools, which showed that the majority of these genes are under purifying selection and are predicted “intolerant” to genetic variations. Rare genetic variants identified in these genes as part of sequencing studies should prompt further in vitro analyses, as they may be involved in the pathogenesis of oligogenic conditions and in the missing heritability of complex disorders.

## DISCLOSURE STATEMENT

The authors declare no conflict of interest.

## Supporting information

Supplementary Material

Supplementary Table
