# Supplementary material for: Properties of human genes guided by their enrichment in rare and common variants
Source: Hum Mutat. 2017 Dec 21;39(3):365–70. doi: 10.1002/humu.23377 (PMC5838408; doi:10.1002/humu.23377)
Supplement: Supplementary file 1 — Supplementary Material [file HUMU-39-365-s001.pdf]

Properties of human genes guided by their enrichment in rare and common variants

Authors: Eman Alhuzimi, Luis G. Leal, Michael J.E. Sternberg, Alessia David

Affiliation: Structural Bioinformatics Group, Department of Life Sciences, Imperial College London, London, SW7 2AZ, UK

SUPPLEMENTARY MATERIAL

Construction of the dataset

Genetic variants occurring in protein coding genes were extracted from ExAC (version 0.3, Release: 13-Jan-2015), UniProt (humsavar.txt, release: 04-Feb-2015) and ClinVar (release:7-Jan-2015). Variants were classified as *'disease-causing'* if a disease association was reported in humsavar.txt or ClinVar. For variants reported in ClinVar, we defined the variant as disease-causing only if it was annotated as "pathogenic". In order to avoid a potential bias, variants annotated as "likely pathogenic" were not included in the analysis. Variants were classified as *'neutral'* when no association with disease was present (variants reported as "polymorphisms" in humsavar.txt and variants from ExAC, not reported as disease-causing in other databases). Non-disease variants were divided according to their global minor allele frequencies (MAF) into: *'rare variants'* (MAF < 0.01) and *'common variants'* (MAF ≥ 0.01). Global MAF data were extracted from Ensembl using the BioMart data-mining tool. We used the global MAF calculated in the ExAC project. For variants not reported in ExAC database we used the global MAF reported in dbSNP (which is calculated from the 1000Genomes project), when available. Variants with no MAF information or reported as "unclassified" in

humsavar.txt, were not included in the analysis. When the gene enrichment analysis (described below) was performed, one gene overlapped between the disease- and rare- EVsets and three genes between the disease- and common- EVsets. In these cases, genes were removed from the rare- and common- EVsets and assigned to the disease-EVset. No overlap was present between the three final gene sets. Disease classification was according to the 10th revision of the International Statistical Classification of Diseases and Related Health Problems (ICD-10) (Brämer, 1988).

PLi scores were obtained from the ExAC database. The dN/dS ratio was calculated according to Ge et al. (Ge et al., 2015) as follows:

$$\frac{dN}{dS} = \frac{N/N\ \text{sites}}{S/S\ \text{sites}}$$

where N and S are the number of observed non-synonymous and synonymous changes in each human gene, respectively, while N and S sites are the expected number of N and S based on the probability of each triplette to mutate to all other possible codons.

The damaging effect of variants was predicted using SIFT, PolyPhen-2, CADD and MSC-corrected CADD scores. All programs were run using default parameters. For SIFT and Polyphen-2 we adopted default thresholds. CADD C-scores range between 0 and 100 and the higher the score, the more likely the variant has a deleterious effect. Although no cut-off is recommended, values ≥10 are at the top 10% of all scores, hence variants with scores ≥10 are less likely to be observed and, therefore, more likely to be deleterious. The gene specific mutation significance cut-offs (MSC) were obtained from http://pec630.rockefeller.edu:8080/MSC/. The MSC was used as a cut-off: variants with CADD scores below the MSC were considered of low impact, whereas variants with CADD scores equal or above the MSC were of high impact (Itan et al., 2016).

Gene-level metrics and gene functional classification

Genes were characterized using the following gene-level metrics with their default parameters: 1) Residual Variation Intolerance Score (RVIS), which is based upon allele frequency and ranks genes according to the gene expected frequency of LoF (Petrovski et al., 2013). A negative score indicates that the query gene has less common functional variation than predicted, thus indicating that the gene is under purifying selection and mutation intolerant; 2) the Excess of De Novo variants (DNE) method (Samocha et al., 2014): the top 1,003 genes that are significantly enriched in de novo LoF were obtained from Samocha et al.; 3) the Gene Damage Index (GDI), which calculates the mutational damage accumulated in the general population for each gene: the less mutated a gene, the more likely it is disease-causing (Itan et al., 2015); 4) the functional indispensability score (Khurana et al., 2013), which is a predicted score built using a model that incorporates gene essentiality, LoF-tolerance, network and evolutionary properties. A median score >0.4 indicates disease-causing genes and genes associated with disease in GWAS. 5) gene selective pressure. This was assessed using the GDI Server, which implements the McDonald-Kreitman neutrality index (Itan et al., 2015).

The DAVID gene functional classification tool (Jiao et al., 2012) was used to explore enrichment in functional categories such as GO terms, pathways (from KEGG, Reactome and Biocarta) and protein domains. A significant enrichment was defined by a Benjamini corrected P value <0.05. The small biological distance was calculated using the human gene connectome (HGC). For each human gene a gene-specific networks is constructed using all human genes sorted on the basis of their predicted biological proximity to a query gene (Itan et al., 2013) (Itan et al., 2014).

Classification of Essential Genes

The Mouse Genome Database (MGD) (Bult et al., 2016) was used to retrieve mouse genes that produce a lethal phenotype. A total of 3,333

mouse genes were classified as essential and could be mapped to human orthologs. Since not all human essential genes have essential mouse orthologs (Liao and Zhang, 2008), the Online GEne Essentiality (OGEE) database (Chen et al., 2012) was also used to identify additional essential genes. The OGEE database includes data for 2,693 experimentally tested human essential genes.

Pathways, Gene Ontology (GO) and protein interactome

Pathways data were extracted from the Reactome Pathway database (Fabregat et al., 2016). GO terms for biological processes, molecular functions and cellular components were retrieved from the GO database (Gene Ontology Consortium, 2015). Protein-protein interactions network data were retrieved from BioGRID (version 3.4.141 (Chatr-Aryamontri et al., 2015).

Statistics

The χ^2 test was used to compare observed and expected frequencies for categorical values. Comparison of medians between two categories was performed using the Mann–Whitney–Wilcoxon test. For comparison between three categories the Kruskal-Wallis Rank Sum Comparison was used to calculate P values. Identification of genes in which disease-causing variants occur more often than expected (genes enriched in disease-causing variants) was done using the hypergeometric test on 17,975 genes in which at least one variant, deleterious or non-deleterious was present. Each gene was assessed against all others. 17,975 p-values were obtained and corrected using the Benjamini-Hochberg procedure (Benjamini and Hochberg, 1995) (total number of tests=17,975). Identification of genes in which rare or common variants occur more often than

expected (genes enriched in rare or common variants), was done using the hypergeometric test on 17,902 genes in which at least one variant, rare or common was present. Each gene was assessed against all others. 17,902 p-values were obtained and corrected using the Benjamini-Hochberg procedure (Benjamini and Hochberg, 1995) (total number of tests=17,902). Results were considered significant if a corrected two-sided P value was <0.05.

Genes enriched in disease-causing variants

Number of genes with at least one disease variant	2,631
Number of genes with at least one non-disease variant	17,902
Number of genes with at least one disease or non-disease variant	17,975
Total number of calculated and corrected p-values	17,975

Genes enriched in rare or common variants

Number of genes with at least one rare variant	17,540
Number of genes with at least one common variant	15,391
Number of genes with at least one rare or common variant	17,902
Total number of calculated and corrected p-values	17,902

SUPPLEMENTARY RESULTS, FIGURES AND TABLES

GO terms and cellular pathways in three gene datasets

In order to obtain a function-driven understanding of the similarities and differences in the genes belonging to the rare-EV and common-EV sets, we mapped these to cellular pathways. Genes enriched in rare variants were more likely ($p<0.01$) to be involved in "signal transduction pathways", similarly to genes enriched in disease-causing variants ("signal transduction", pathways" and "metabolism"), whereas genes enriched in common variants were annotated as involved in "immune system pathway" ($p<0.01$). We also categorized each gene in the three sets by using the Gene Ontology (GO) classification (Gene Ontology Consortium, 2015). Genes in the disease-EVset and rare-EVset were again significantly ($p<0.05$) more likely to be involved in core biological processes (namely "metabolic process" and "biological regulation" for genes in the disease-EVset and "cellular process", "biogenesis" and "catalytic activity" genes in the rare-EVset) compared to genes in the common-EVset when GO terms were examined. Nevertheless, genes in the common-EVset were more likely to be involved in " cellular components", biological adhesions" and "developmental and cellular processes" compared to the disease-EVset.

Supp. Figure S1 The dN/dS ratio in the three gene enriched sets. Box plot depicts median and 1st (Q1) and 3rd quartiles (Q3); whiskers denotes the Q3+/-1.5 *IQR. P value <0.0001 (Kruskal-Wallis Rank Sum test).

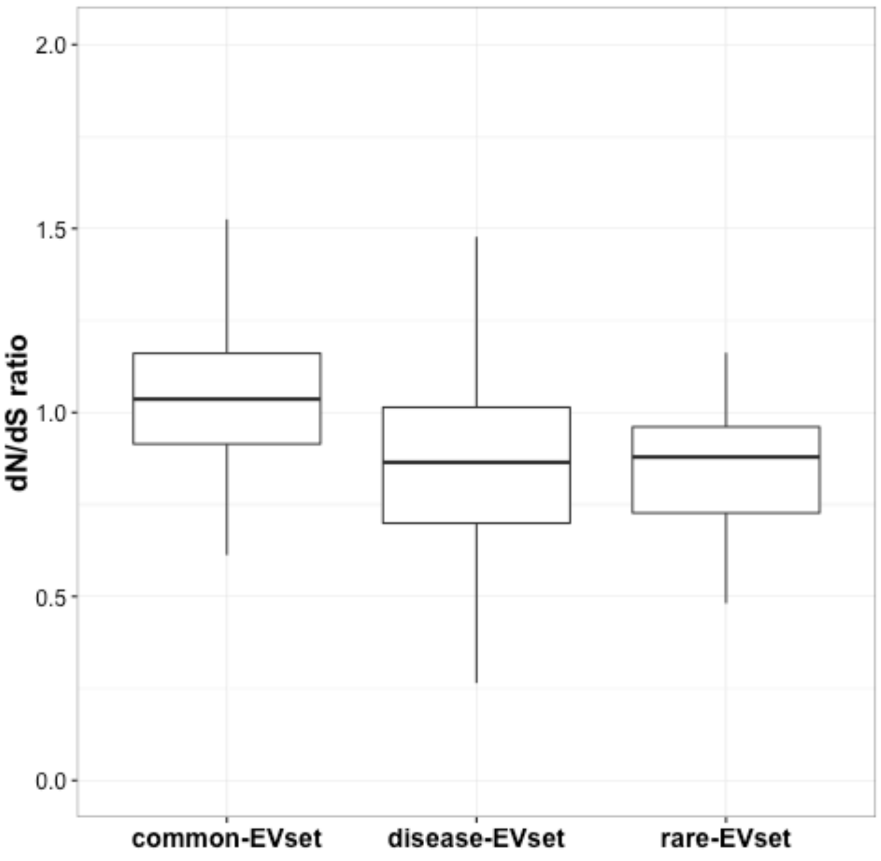

Supp. Figure S2 CADD C-scores for missense and nonsense variants in 12 genes enriched in rare variants.

The violin plots show the median C-scores for A) missense and B) nonsense (stop-gained) variants.

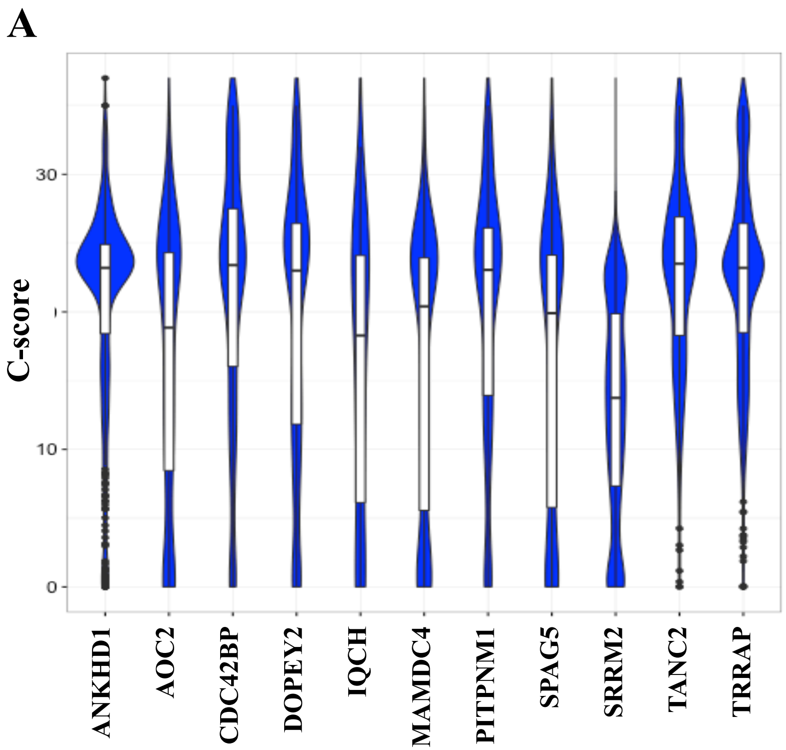

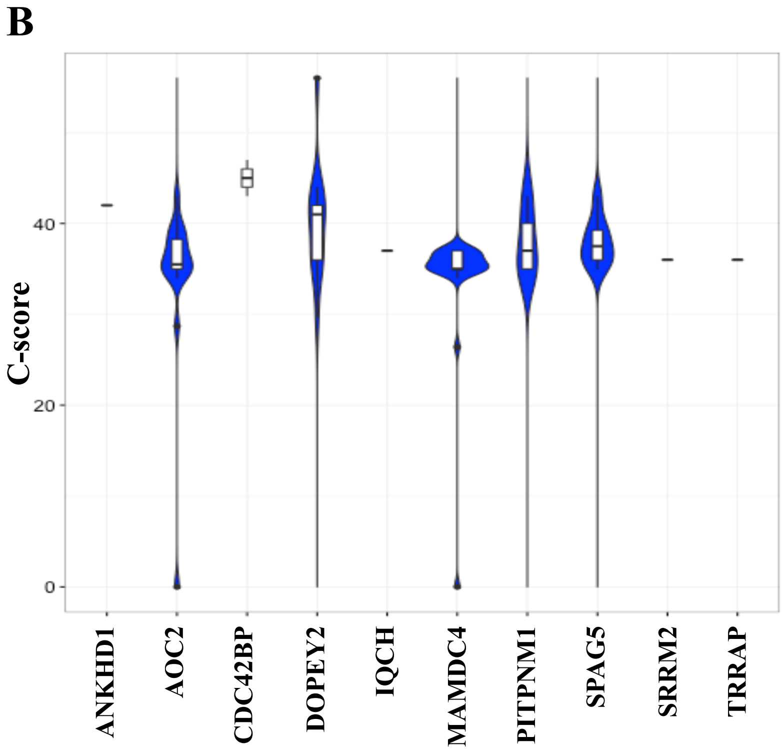

Supp. Figure S3 Percentage of specific and non-specific genetic variants in 12 genes enriched in rare variants across different populations.

AFR, African/African American; AMR, Latino; ASJ, Ashkenazi Jewish; EAS, East Asian; FIN, Finnish; NFE, Non-Finnish European; SAS, South Asian; OTH, Other. "All pops", variant is present in all population; "+2 pops.", variant is present in 2 or more populations.

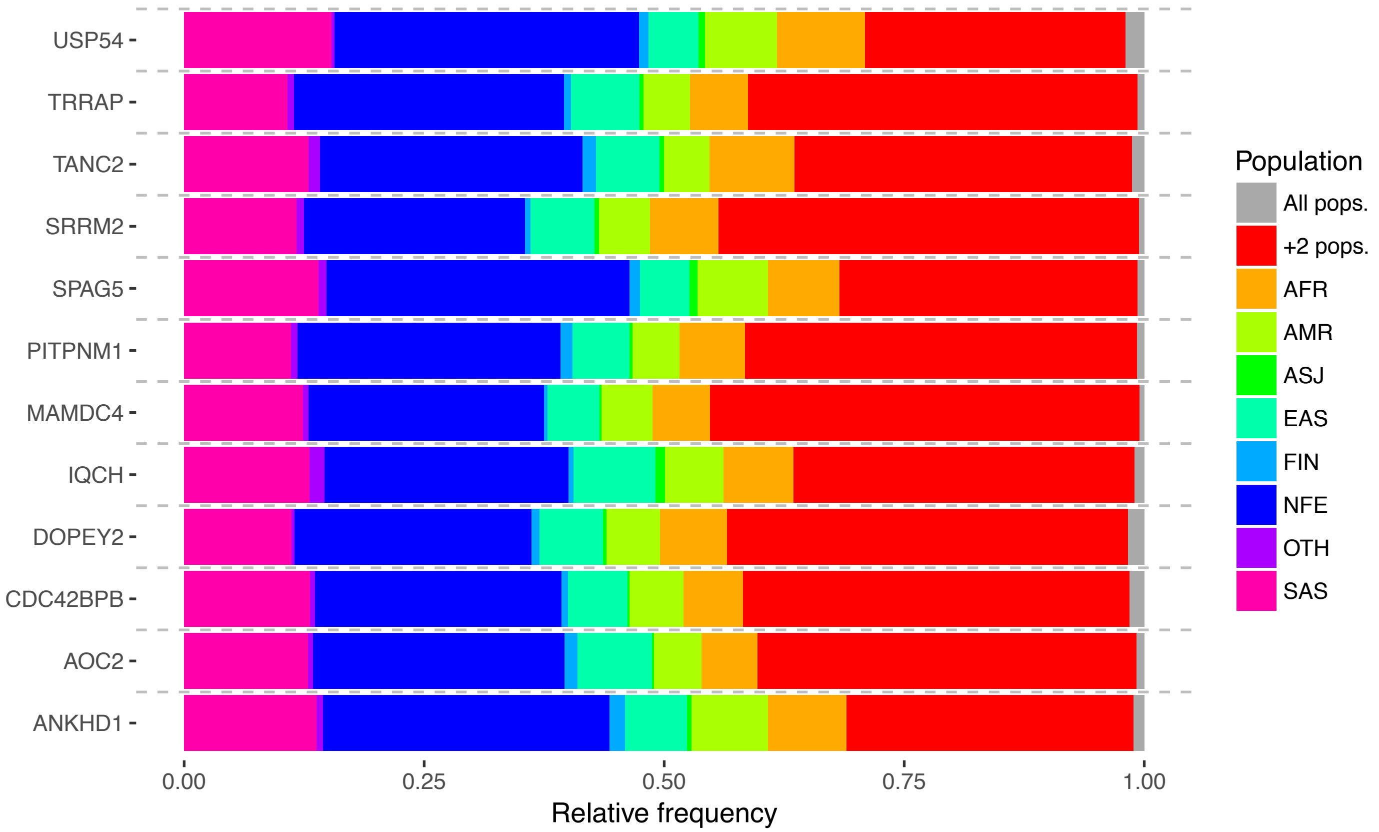

Supp. Figure S4 Percentage of common and rare variants per population and gene.

AFR, African/African American; AMR, Latino; ASJ, Ashkenazi Jewish; EAS, East Asian; FIN, Finnish; NFE, Non-Finnish European; SAS, South Asian; OTH, Other. "All pops", variant is present in all population; "+2 pops.", variant is present in 2 or more populations.

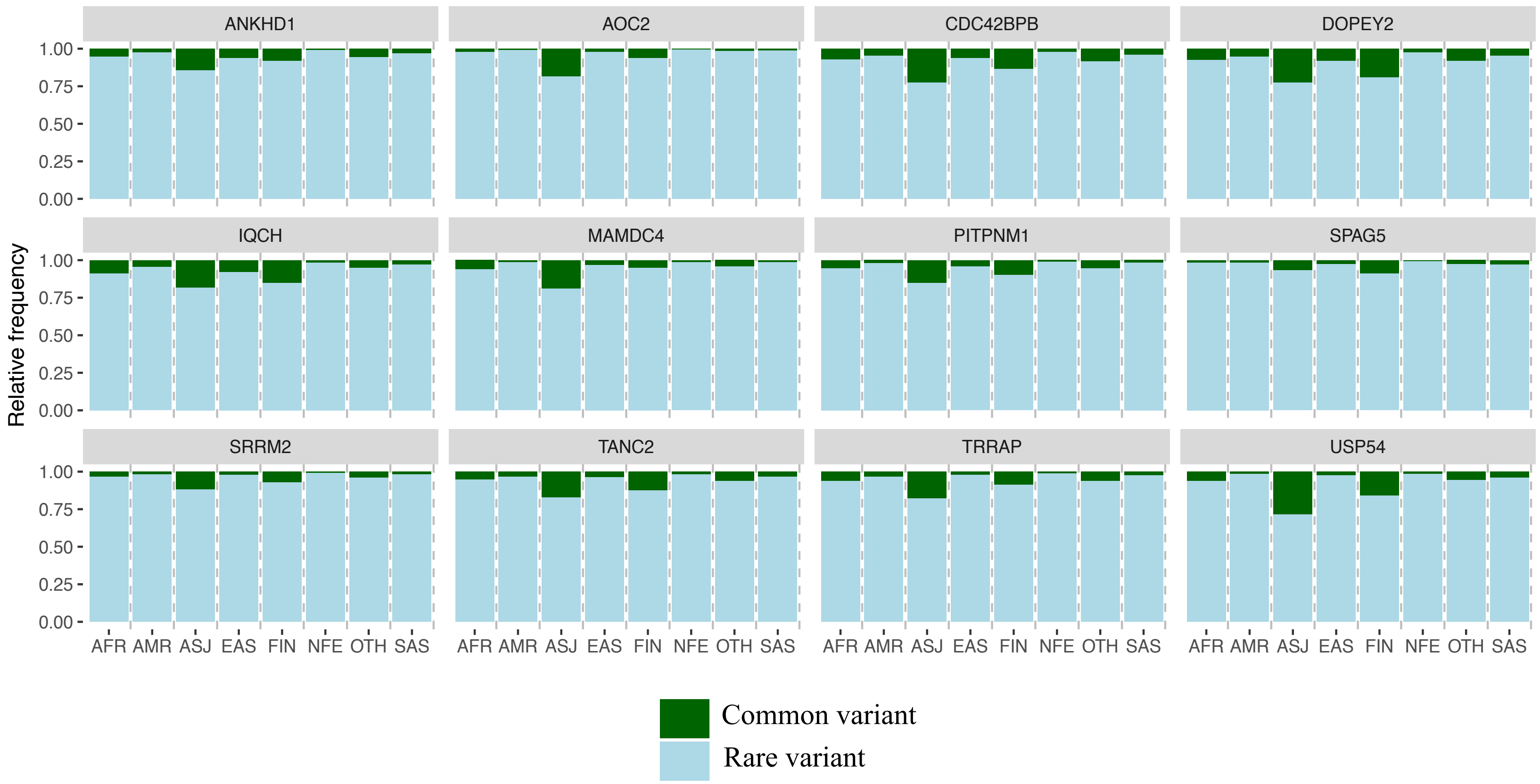

Supp. Table S1 pLi scores in the three gene enriched sets. Data are presented as mean and standard error (SE). P <0.0001 (Kruskal-Wallis Rank Sum test).

	**common-EVset**	**rare-EVset**	**disease-EVset**
**Mean**	**0.11**	**0.48**	**0.36**
**SE**	**0.02**	**0.02**	**0.09**

Supp. Table S2A Genes were considered essential if reported as such in the OGEE database or if human orthologs of mouse essential genes.

	**Essential**	**Non-Essential**	**Total**	**P value ***
**Disease-EVset**	511	289	800	0.22
**Rare-EVset**	17	15	32	
**Total**	528	304	832	

*, Corrected P value. Total n. of tests =4 (see methods in Supplementary Material)

Supp. Table S2B Genes were considered essential if reported as such in OGEE database or if human orthologs of mouse essential genes.

	Essential	Non-Essential	Total	P value *
**Rare-EVset**	17	15	32	<0.01
**Common-EVset**	30	252	282	
**Total**	47	267	314	

*, Corrected P value. Total n. of tests =4 (see methods in Supplementary Material)

Supp. Table S2C Genes were considered essential if reported as such in OGEE database or if human orthologs of mouse essential genes.

	Essential	Non-Essential	Total	P value *
**Disease-EVset**	511	289	800	<0.01
**Rare&Common EVset**	47	267	314	
**Total**	558	556	1114	

*, Corrected P value. Total n. of tests =4 (see methods in Supplementary Material)

Supp. Table S2D Genes were considered essential if reported as such in OGEE database or if human orthologs of mouse essential genes.

	Essential	Non-Essential	Total	P value *
**Disease-EVset**	511	289	800	<0.01
**Common-EVset**	30	252	282	
**Total**	558	556	1114	

*, Corrected P value. Total n. of tests =4 (see methods in Supplementary Material)

Supp. Table S3 Genes with short variants annotated as disease-causing or with a significant association with disease reported in the GWAS catalog.

	n. of genes (%)	Total n. of genes	P value
Rare_EVset	20 (62.5%)	32	<0.001
Common_EVset	91 (32.3%)	282	

Supp. Table S4 HGMD and DECIPHER variant annotations for 12 genes enriched in rare variants. For HGMD professional, only information available from the public version of the database is reported.

Gene name	DECIPHER		HGMD public	HGMD professional
	**Copy number variations (phenotype)**	**Sequence variants (phenotype)**	**Variant (associated phenotype)**	**Variant**
AOC2	Copy number gain and loss (Congenital abnormalities)	0	0	0
MAMDC4	Copy number gain and loss (Congenital abnormalities)	0	0	n.a.
ANKHD1	Copy number gain and loss (Congenital abnormalities)	0	0	0
CDC42BPB	Copy number gain and loss (Congenital abnormalities)	0	1 nonsense (Autism?);1 gross deletion (Autism spectrum disorder); 1 splicing (associated with diffuse large B-cell lymphoma);	2 missense/nonsense; 1 splicing; 1 gross insertion/deletion
SPAG5	Copy number loss (Cerebral palsy, intellectual disability, Polymicrogyria, Tetraplegia)	0	0	n.a.
TRRAP	Copy number gain and loss (Congenital abnormalities)	0	2 missense (Schizophrenia?, Autism?); 1 splicing (Schizophrenia?); 1 gross duplication (Li-Fraumeni syndrome)	10 missense/nonsense; 1 splicing; 1 gross insertion/deletion

TANC2	Copy number gain and loss (Congenital abnormalities)	* p.Gly1280Val (Coloboma)	1 missense (Intellectual disability?)	2 missense/nonsense
IQCH	Copy number gain and loss (n.a.)	0	0	0
USP54	Copy number gain and loss (Congenital abnormalities)	0	1 small insertion (Autism spectrum disorder?)	1 small insertion
SRRM2	Copy number gain and loss (Congenital abnormalities, seizures)	** p.Lys1781AsnfsTer9 (n.a.)	0	n.a.
DOPEY2	Copy number gain and loss (Congenital abnormalities)	0	0	0
PITPNM1	Copy number gain and loss (Congenital abnormalities)	0	0	n.a.

* , de-novo heterozygous variant – clinical significance unknown , ** , de-novo variant – clinical significance unknown; n.a., not available.

Supp. Table S5 Twelve genes enriched in rare variants (rare-EVset) that have no short genetic variants reported to be associated with disease in OMIM, UniProt, ClinVar or the GWAS Catalog (large deletions and insertions >50Kb were not included in the analysis). pLi scores were extracted from the ExAC database. A pLi score ≥0.9 is indicative of the gene extreme intolerance to loss of function variations.

Gene symbol	Gene name	pLI score	Function *
ANKHD1	Ankyrin repeat and KH domain-containing protein 1	1.00	This gene may play a role as a scaffolding protein that may be associated with the abnormal phenotype of leukaemia cells. Isoform 2 may possess an antiapoptotic effect and protect cells during normal cell survival through its regulation of caspases. [PMID:16098192]
CDC42BPB	Serine/threonine-protein kinase MRCK beta	1.00	This serine/threonine-protein kinase is an important downstream effector of CDC42 and plays a role in the regulation of cytoskeleton reorganization and cell migration. It regulates actin cytoskeletal reorganization via phosphorylation of PPP1R12C and MYL9/MLC2. In concert with MYO18A and LURAP1, it is involved in modulating lamellar actomyosin retrograde flow that is crucial to cell protrusion and migration. It phosphorylates PPP1R12A. [PMID:18854160, PMID:21457715, PMID:21949762]
TRRAP	Transformation/transcription domain-associated protein	1.00	An adapter protein found in various multiprotein chromatin complexes with histone acetyltransferase activity (HAT). It gives a specific tag for epigenetic transcription activation. Component of the NuA4 histone acetyltransferase complex, which is responsible for acetylation of nucleosomal histones H4 and H2A. This gene plays a central role in MYC transcription activation, and also participates in cell transformation by MYC. It is required for p53/TP53-, E2F1- and E2F4-mediated transcription activation. Also involved in transcription activation mediated by the adenovirus E1A, a viral oncoprotein that deregulates transcription of key genes. This gene may be required for the mitotic checkpoint and normal cell cycle progression. It is also a component of a SWR1-like complex that specifically mediates the removal of histone H2A.Z/H2AFZ from the nucleosome. [PMID:11418595, PMID:12138177, PMID:12660246, PMID:12743606, PMID:14966270, PMID:17967892, PMID:24463511, PMID:9708738]
TANC2	Tetratricopeptide repeat, ankyrin repeat and coiled-coil domain-containing protein 2	1.00	This gene may play a role in embryonic development.[PMID:21068316]

PITPNM1	Membrane-associated phosphatidylinositol transfer protein 1	0.72	This gene regulates RHOA activity, and plays a role in cytoskeleton remodeling. Necessary for normal completion of cytokinesis. Plays a role in maintaining normal diacylglycerol levels in the Golgi apparatus. Binds phosphatidyl inositol phosphates (in vitro). May catalyse the transfer of phosphatidylinositol and phosphatidylcholine between membranes (By similarity). Necessary for maintaining the normal structure of the endoplasmic reticulum and the Golgi apparatus. Required for protein export from the endoplasmic reticulum and the Golgi. Binds calcium ions. [PMID:10022914, PMID:11909959, PMID:15545272, PMID:15723057]
AOC2	Retina-specific copper amine oxidase	0.00	This gene has a monoamine oxidase activity with substrate specificity for 2-phenylethylamine and tryptamine. May play a role in adipogenesis. It may be a critical modulator of signal transmission in retina. [PMID:17400359, PMID:19588076].
MAMDC4	Apical endosomal glycoprotein	0.00	This is gene may be involved in the sorting and selective transport of receptors and ligands across polarized epithelia.
SPAG5	Sperm-associated antigen 5	0.00	This gene is an essential component of the mitotic spindle required for normal chromosome segregation and progression into anaphase. In non-mitotic cells, upon stress induction, this gene inhibits mammalian target of rapamycin complex 1 (mTORC1) association and recruits the mTORC1 component RPTOR to stress granules (SGs), thereby preventing mTORC1 hyperactivation-induced apoptosis. It may also enhance GSK3B-mediated phosphorylation of other substrates, such as MAPT/TAU. [PMID:12356910, PMID:17664331, PMID:18055457, PMID:18361916, PMID:21402792, PMID:23953116, PMID:26297806, PMID:11724960]
IQCH	IQ domain-containing protein H / Testis development protein NYD-SP5	0.00	This gene may play a regulatory role in spermatogenesis. [PMID:15897968]
USP54	Inactive ubiquitin carboxyl-terminal hydrolase 54	0.00	This gene is a member of the ubiquitin-specific protease (USP) family. It has no peptidase activ [PMID:14715245]
SRRM2	Serine/arginine repetitive matrix protein 2	n.a.	This gene is involved in pre-mRNA splicing. May function at or prior to the first catalytic step of splicing at the catalytic centre of the spliceosome. May do so by stabilizing the catalytic centre or the position of the RNA substrate (By similarity). Binds to RNA.[PMID:10668804]
DOPEY2	Protein dopey-2	0.00	This gene may be involved in protein traffic between late Golgi and early endosomes. It is overexpressed in lymphoblasts from Down syndrome patients. [PMID:12767918]

n.a., not available. *, Function description was adapted from the UniProt database.

Supp. Table S6 *In silico* predictions for missense variants by SIFT, Polyphen2 and MSC-corrected CADD scores. Variants are reported "predicted damaging" if above the default SIFT score, if assigned to "probably" or "possibly damaging" by PolyPhen-2 or if the CADD score was equal or above the gene specific MSC.

	SIFT			PolyPhen-2			MSC-CADD scores		
**GENE**	**Total n.**	**Predicted damaging**	**(%)**	**Total n.**	**Predicted damaging**	**(%)**	**Total n.**	**Predicted damaging**	**(%)**
ANKHD1	525	310	59.05	525	266	50.67	525	312	59.43
AOC2	350	211	60.29	350	200	57.14	350	143	40.86
CDC42BPB	432	189	43.75	432	144	33.33	432	246	56.94
DOPEY2	821	385	46.89	821	403	49.09	821	424	51.64
IQCH	31	18	58.06	31	17	54.84	31	11	35.48
MAMDC4	417	202	48.44	417	189	45.32	417	170	40.77
PITPNM1	369	180	48.78	369	183	49.59	369	193	52.30
SPAG5	246	109	44.31	246	99	40.24	246	96	39.02
SRRM2	1517	1114	73.43	1517	725	47.79	1517	171	11.27
TANC2	348	180	51.72	348	200	57.47	348	200	57.47
TRRAP	747	290	38.82	747	287	38.42	747	419	56.09
USP54	0	0	0.00	0	0	0.00	0	0	0.00

Total n., total number of missense variants analysed

Predictor tools available at: MSC at http://lab.rockefeller.edu/casanova/MSC; SIFT at http://sift.jcvi.org/; PolyPhen2 at http://genetics.bwh.harvard.edu/pph2/

Supp. Table S7 Description of 18 genetic variants with allele frequencies changing from rare to common across different populations.

Gene	Reference Id	Impact	Aa	Aa	In silico prediction (score)			gnomAD							
		on protein	position	change	SIFT	PolyPhen	CADD	AFR_AF	AMR_AF	ASJ_AF	EAS_AF	FIN_AF	NFE_AF	OTH_AF	SAS_AF
ANKHD1	rs61758138	missense	143	Q/E	D (0) *	Probably D (0.93)	23.8	0.0009569	0.001526	**0.01011**	6.24E-05	**0.01076**	0.007133	0.00655	0.0005223
AOC2	rs536168385	missense	749	P/T	D (0.01)	Possibly D (0.626)	23.6	0.0001961	0	0	0	0	2.70E-05	0.00147	**0.02105**
AOC2	rs35833794	missense	141	P/L	D (0)	Probably D (1)	27.3	**0.01587**	0.001668	0	0	0	9.04E-05	0.001467	9.75E-05
AOC2	rs34351794	missense	427	E/D	D (0)	Probably D (1)	23.7	0	0	0	**0.01345**	0	0	0.0001823	0
AOC2	rs34625494	missense	692	T/I	D (0)	Probably D (1)	32	**0.01555**	0.0002382	0	0	0	0	0.0005468	6.50E-05
DOPEY2	rs117132686	missense	272	S/Y	D (0)	Possibly D (0.663)	23.2	0	8.94E-05	0.0002034	**0.02703**	0	0	0.001095	0.0002275
DOPEY2	rs142091518	missense	188	S/N	D (0)	Probably D (0.95)	25.5	0.0006557	**0.07786**	0	0.0001168	0	1.81E-05	0.00942	0
DOPEY2	rs143714922	missense	1938	R/H	D (0)	Probably D (0.971)	34	0.0007188	**0.07273**	0	0	0.000269	0.0001522	0.006565	0
MAMDC4	rs186097368	missense/splice	53	Y/N	D (0)	Possibly D (0.66)	15.96	0.0001321	0.0001804	0	**0.02261**	0	9.20E-06	0.001495	0.0003304
MAMDC4	rs140559332	missense	780	V/G	D (0.02)	Possibly D (0.881)	26	0	0.0001192	**0.03067**	0	0	0.0005067	0.00257	0.0001625
MAMDC4	rs202115673	missense	387	R/Q	D (0.02)	Probably D (0.985)	27.4	0	0.0001667	**0.01059**	0	0	5.64E-05	0.0004134	3.45E-05
MAMDC4	rs376290390	missense	487	E/K	D (0)	Probably D (0.992)	27.1	0	3.53E-05	0	0	0	1.21E-05	0.0002228	**0.01032**
SPAG5	rs143024358	missense	1082	A/V	D (0.04)	Probably D (0.946)	24.8	0.0007846	0.0007743	0.0002031	0	**0.01048**	0.003593	0.003654	0.00757
SPAG5	rs145549199	start lost	1	M/V	D (0) *	Probably D (0.969)	25.3	0.0001961	0.0008934	0.00132	5.80E-05	**0.03154**	0.00369	0.003281	**0.01234**
SRRM2	rs117133016	missense	2033	R/P	D (0) *	Probably D (0.915)	22.3	0.001178	0.001221	0.0007109	0.000116	0.006146	**0.01198**	0.004196	0.0003249
SRRM2	rs114899013	missense	435	S/T	D (0) *	Probably D (0.931)	8.843	**0.01407**	0.0008342	0	0	0	4.48E-05	0.0007302	0.000195
SRRM2	rs138495768	missense	2739	P/S	D (0) *	Probably D (0.994)	20.9	0.0008401	0.001987	0.004785	0.0001844	0	0.003808	0.009353	**0.03923**
SRRM2	rs114848780	missense	1752	R/P	D (0.03) *	Probably D (0.995)	22.7	**0.0519**	0.00271	0.000203	0	0	6.27E-05	0.002007	9.75E-05

*, low confidence; D, deleterious; CADD, CADD C-score;

AFR, African/African American; AMR, Latino; ASJ, Ashkenazi Jewish; EAS, East Asian; FIN, Finnish; NFE, Non-Finnish European; OTH, Other; SAS, South Asian.

Supp. Table S8 Small biological distance between the 12 genes enriched in rare variants calculated using the human gene connectome (available at http://lab.rockefeller.edu/casanova/GDI).

'Distance', small biological distance; 'Rank', ranking of the target gene compared to all human genes in the query gene specific connectome; 'BRP', best reciprocal *p*-value or smallest of the mutual *p*-values between the query and target gene; 'Median ratio' and 'Average ratio', the median and average distance between the query gene and all human genes; 'Sphere', the sphere of the target gene around the query; 'Degrees of separation', the number of nodes between the query and target genes. For a comprehensive explanation of each term please refer to Itan et al. (Itan et al., 2014).

Query Gene	Target gene	Distance	Rank	P-Value	BRP	Median ratio	Average ratio	Sphere	Degrees of Separation
PITPNM1	TRRAP	10.41667	1285	0.07678	0.36237	0.69444	0.67358	3	3
PITPNM1	SPAG5	10.83333	4683	0.2798	0.14859	0.72222	0.70052	5	3
PITPNM1	SRRM2	18.38299	11537	0.68931	0.57149	1.22553	1.18871	6	4
PITPNM1	TANC2	18.82075	11717	0.70007	0.21402	1.25472	1.21702	6	3
PITPNM1	ANKHD1	19.44444	13093	0.78228	0.6218	1.2963	1.25735	7	4
PITPNM1	USP54	21.01901	14377	0.859	0.61313	1.40127	1.35917	7	4
PITPNM1	CDC42BPB	21.38889	14577	0.87094	0.64749	1.42593	1.38309	7	4
PITPNM1	DOPEY2	22.29585	15007	0.89664	0.61923	1.48639	1.44173	7	4
PITPNM1	MAMDC4	24.70085	15314	0.91498	0.31535	1.64672	1.59725	7	4
PITPNM1	AOC2	44.79755	16650	0.9948	0.60937	2.9865	2.89678	7	5
PITPNM1	IQCH	N/A	N/A	N/A	N/A	N/A	N/A	N/A	N/A
MAMDC4	ANKHD1	24.02027	3451	0.20619	0.82004	0.88964	0.79467	4	3
MAMDC4	CDC42BPB	24.02027	4645	0.27753	0.84161	0.88964	0.79467	5	3
MAMDC4	PITPNM1	24.70085	5278	0.31535	0.91498	0.91485	0.81718	5	4

MAMDC4	TRRAP	26.34995	5777	0.34516	0.96594	0.97592	0.87174	5	4
MAMDC4	SPAG5	27.95796	8543	0.51043	0.9372	1.03548	0.92494	6	4
MAMDC4	USP54	30.08808	10032	0.59939	0.88869	1.11437	0.99541	6	4
MAMDC4	DOPEY2	31.36492	10842	0.64779	0.87698	1.16166	1.03765	6	4
MAMDC4	SRRM2	35.84048	11950	0.71399	0.98076	1.32743	1.18572	6	5
MAMDC4	TANC2	38.46812	13986	0.83563	0.94264	1.42475	1.27265	7	4
MAMDC4	AOC2	69.14168	16652	0.99492	0.97359	2.5608	2.28742	7	6
MAMDC4	IQCH	N/A	N/A	N/A	N/A	N/A	N/A	N/A	N/A
USP54	ANKHD1	16.50901	3635	0.21718	0.35747	0.82545	0.7975	4	3
USP54	CDC42BPB	18.81606	5789	0.34588	0.43473	0.9408	0.90895	5	3
USP54	TRRAP	20.46346	7242	0.43269	0.91331	1.02317	0.98853	5	4
USP54	PITPNM1	21.01901	10262	0.61313	0.859	1.05095	1.01537	6	4
USP54	SPAG5	21.01901	10564	0.63118	0.83259	1.05095	1.01537	6	4
USP54	SRRM2	22.02502	11763	0.70281	0.86222	1.10125	1.06397	6	4
USP54	DOPEY2	24.42597	13913	0.83127	0.73585	1.2213	1.17995	7	4
USP54	MAMDC4	30.08808	14874	0.88869	0.59939	1.5044	1.45347	7	4
USP54	TANC2	30.91069	14960	0.89383	0.82117	1.54553	1.49321	7	4
USP54	AOC2	47.46021	16560	0.98942	0.75342	2.37301	2.29267	7	5
USP54	IQCH	N/A	N/A	N/A	N/A	N/A	N/A	N/A	N/A
SPAG5	PITPNM1	10.83333	2487	0.14859	0.2798	0.63725	0.65661	4	3
SPAG5	TRRAP	12.35574	5080	0.30352	0.61923	0.72681	0.74889	5	3
SPAG5	SRRM2	17.82744	9659	0.5771	0.49752	1.04867	1.08053	6	4
SPAG5	ANKHD1	18.88889	11634	0.69511	0.54233	1.11111	1.14486	6	4
SPAG5	USP54	21.01901	13935	0.83259	0.63118	1.23641	1.27397	7	4
SPAG5	CDC42BPB	21.38889	14120	0.84364	0.6592	1.25817	1.29639	7	4
SPAG5	DOPEY2	22.29585	14568	0.87041	0.63829	1.31152	1.35136	7	4
SPAG5	MAMDC4	27.95796	15686	0.9372	0.51043	1.64459	1.69454	7	4
SPAG5	TANC2	28.42767	15738	0.94031	0.70915	1.67222	1.72301	7	4
SPAG5	AOC2	44.10311	16628	0.99349	0.52082	2.5943	2.67311	7	5
SPAG5	IQCH	N/A	N/A	N/A	N/A	N/A	N/A	N/A	N/A

TANC2	ANKHD1	13.37793	769	0.04595	0.25482	0.55741	0.54286	2	2
TANC2	CDC42BPB	13.40037	957	0.05718	0.23744	0.55835	0.54377	3	2
TANC2	PITPNM1	18.82075	3582	0.21402	0.70007	0.7842	0.76372	4	3
TANC2	SRRM2	19.15859	3850	0.23003	0.64868	0.79827	0.77743	4	3
TANC2	TRRAP	20.42813	5599	0.33453	0.91175	0.85117	0.82895	5	3
TANC2	SPAG5	28.42767	11869	0.70915	0.94031	1.18449	1.15356	6	4
TANC2	USP54	30.91069	13744	0.82117	0.89383	1.28795	1.25431	7	4
TANC2	DOPEY2	32.11241	14473	0.86473	0.88289	1.33802	1.30308	7	4
TANC2	MAMDC4	38.46812	15777	0.94264	0.83563	1.60284	1.56099	7	4
TANC2	AOC2	57.41548	16636	0.99397	0.89956	2.39231	2.32984	7	5
TANC2	IQCH	N/A	N/A	N/A	N/A	N/A	N/A	N/A	N/A
SRRM2	TRRAP	10.03724	1500	0.08962	0.27478	0.59043	0.60445	3	3
SRRM2	SPAG5	17.82744	8327	0.49752	0.5771	1.04867	1.07358	5	4
SRRM2	PITPNM1	18.38299	9565	0.57149	0.68931	1.08135	1.10704	6	4
SRRM2	ANKHD1	18.91372	10438	0.62365	0.55099	1.11257	1.139	6	4
SRRM2	TANC2	19.15859	10857	0.64868	0.23003	1.12698	1.15375	6	3
SRRM2	CDC42BPB	21.77789	14188	0.8477	0.69881	1.28105	1.31148	7	4
SRRM2	USP54	22.02502	14431	0.86222	0.70281	1.29559	1.32637	7	4
SRRM2	DOPEY2	23.30186	14793	0.88385	0.67348	1.3707	1.40326	7	4
SRRM2	MAMDC4	35.84048	16415	0.98076	0.71399	2.10826	2.15835	7	5
SRRM2	AOC2	43.84978	16611	0.99247	0.43461	2.5794	2.64067	7	5
SRRM2	IQCH	N/A	N/A	N/A	N/A	N/A	N/A	N/A	N/A
DOPEY2	ANKHD1	19.19724	4459	0.26642	0.55805	0.91415	0.86382	5	3
DOPEY2	CDC42BPB	19.77369	6383	0.38137	0.44835	0.9416	0.88976	5	3
DOPEY2	TRRAP	21.74029	7982	0.47691	0.9393	1.03525	0.97825	5	4
DOPEY2	PITPNM1	22.29585	10364	0.61923	0.89664	1.06171	1.00325	6	4
DOPEY2	SPAG5	22.29585	10683	0.63829	0.87041	1.06171	1.00325	6	4
DOPEY2	SRRM2	23.30186	11272	0.67348	0.88385	1.10961	1.04852	6	4
DOPEY2	USP54	24.42597	12316	0.73585	0.83127	1.16314	1.0991	6	4

DOPEY2	MAMDC4	31.36492	14678	0.87698	0.64779	1.49357	1.41133	7	4
DOPEY2	TANC2	32.11241	14777	0.88289	0.86473	1.52916	1.44497	7	4
DOPEY2	AOC2	49.05625	16537	0.98805	0.79429	2.33601	2.20739	7	5
DOPEY2	IQCH	N/A	N/A	N/A	N/A	N/A	N/A	N/A	N/A
ANKHD1	TRRAP	12.1875	2962	0.17697	0.58654	0.67708	0.65098	4	3
ANKHD1	TANC2	13.37793	4265	0.25482	0.04595	0.74322	0.71456	5	2
ANKHD1	USP54	16.50901	5983	0.35747	0.21718	0.91717	0.88181	5	3
ANKHD1	SPAG5	18.88889	9077	0.54233	0.69511	1.04938	1.00892	6	4
ANKHD1	SRRM2	18.91372	9222	0.55099	0.62365	1.05076	1.01025	6	4
ANKHD1	DOPEY2	19.19724	9340	0.55805	0.26642	1.06651	1.02539	6	3
ANKHD1	PITPNM1	19.44444	10407	0.6218	0.78228	1.08025	1.0386	6	4
ANKHD1	CDC42BPB	21.38889	12277	0.73352	0.62329	1.18827	1.14246	6	4
ANKHD1	MAMDC4	24.02027	13725	0.82004	0.20619	1.33446	1.28301	7	3
ANKHD1	AOC2	44.79755	16541	0.98829	0.56988	2.48875	2.3928	7	5
ANKHD1	IQCH	N/A	N/A	N/A	N/A	N/A	N/A	N/A	N/A
TRRAP	SRRM2	10.03724	4599	0.27478	0.08962	0.91248	0.80909	5	3
TRRAP	PITPNM1	10.41667	6065	0.36237	0.07678	0.94697	0.83967	5	3
TRRAP	ANKHD1	12.1875	9817	0.58654	0.17697	1.10795	0.98242	6	3
TRRAP	CDC42BPB	12.29167	10225	0.61092	0.11018	1.11742	0.99082	6	3
TRRAP	SPAG5	12.35574	10364	0.61923	0.30352	1.12325	0.99598	6	3
TRRAP	TANC2	20.42813	15260	0.91175	0.33453	1.8571	1.64669	7	3
TRRAP	USP54	20.46346	15286	0.91331	0.43269	1.86031	1.64953	7	4
TRRAP	DOPEY2	21.74029	15721	0.9393	0.47691	1.97639	1.75246	7	4
TRRAP	MAMDC4	26.34995	16167	0.96594	0.34516	2.39545	2.12404	7	4
TRRAP	AOC2	44.10311	16696	0.99755	0.46018	4.00937	3.5551	7	5
TRRAP	IQCH	N/A	N/A	N/A	N/A	N/A	N/A	N/A	N/A
CDC42BPB	TRRAP	12.29167	1844	0.11018	0.61092	0.61458	0.64872	4	3
CDC42BPB	TANC2	13.40037	3974	0.23744	0.05718	0.67002	0.70724	4	2
CDC42BPB	USP54	18.81606	7276	0.43473	0.34588	0.9408	0.99306	5	3

CDC42BPB	DOPEY2	19.77369	7504	0.44835	0.38137	0.98868	1.0436	5	3
CDC42BPB	ANKHD1	21.38889	10432	0.62329	0.73352	1.06944	1.12885	6	4
CDC42BPB	PITPNM1	21.38889	10837	0.64749	0.87094	1.06944	1.12885	6	4
CDC42BPB	SPAG5	21.38889	11033	0.6592	0.84364	1.06944	1.12885	6	4
CDC42BPB	SRRM2	21.77789	11696	0.69881	0.8477	1.08889	1.14938	6	4
CDC42BPB	MAMDC4	24.02027	14086	0.84161	0.27753	1.20101	1.26773	7	3
CDC42BPB	AOC2	47.22811	16570	0.99002	0.72373	2.36141	2.49258	7	5
CDC42BPB	IQCH	N/A	N/A	N/A	N/A	N/A	N/A	N/A	N/A
AOC2	SRRM2	43.84978	7274	0.43461	0.99247	0.99659	1.02149	5	5
AOC2	SPAG5	44.10311	8717	0.52082	0.99349	1.00234	1.02739	6	5
AOC2	TRRAP	44.10311	7702	0.46018	0.99755	1.00234	1.02739	5	5
AOC2	ANKHD1	44.79755	9538	0.56988	0.98829	1.01813	1.04357	6	5
AOC2	PITPNM1	44.79755	10199	0.60937	0.9948	1.01813	1.04357	6	5
AOC2	CDC42BPB	47.22811	12113	0.72373	0.99002	1.07337	1.10019	6	5
AOC2	USP54	47.46021	12610	0.75342	0.98942	1.07864	1.10559	7	5
AOC2	DOPEY2	49.05625	13294	0.79429	0.98805	1.11491	1.14277	7	5
AOC2	TANC2	57.41548	15056	0.89956	0.99397	1.3049	1.3375	7	5
AOC2	MAMDC4	69.14168	16295	0.97359	0.99492	1.5714	1.61067	7	6
AOC2	IQCH	N/A	N/A	N/A	N/A	N/A	N/A	N/A	N/A

N/A, not available.

Supp. Table S9 Gene metrics for 12 genes enriched in rare variants. RVIS, GDI and selective pressure scores are presented.

GDI and selective pressure prediction tool are available at http://lab.rockefeller.edu/casanova/GDI; RVIS at http://genic-intolerance.org/.

	RVIS		GDI		Selective pressure	
**GENE**	**Score**	**Percentile**	**Score**	**Prediction**	**McDonald-Kreitman neutrality index**	**Prediction**
AOC2	-0.12	44.1	237.89	Medium	0.53395	Moderate purifying
MAMDC4	1.89	97.28	675.67	Medium	1.46743	Moderate positive
ANKHD1	-2.36	1.14	265.86	Medium	1.29205	Moderate positive
CDC42BPB	-3.29	0.42	159.11	Medium	0.06814	Moderate purifying
SPAG5	-0.39	27.08	276.44	Medium	0.90217	Moderate purifying
TRRAP	-6.14	0.04	222.00	Medium	0.02488	Moderate purifying
TANC2	-2.78	0.67	191.80	Medium	0.12843	Moderate purifying
IQCH	-0.42	25.84	166.12	Medium	0.54036	Moderate purifying
USP54	1.06	91.38	558.06	Medium	0.59861	Moderate purifying
SRRM2	-4.51	0.08	2273.47	Medium	0.7297	Moderate purifying
DOPEY2	-3.65	0.28	3302.00	Medium	0.16865	Moderate purifying
PITPNM1	-1.01	8.20	187.25	Medium	0.13039	Moderate purifying

References

Benjamini Y, Hochberg Y. 1995. Controlling the false discovery rate: a practical and powerful approach to multiple testing. J R Stat Soc Ser B Methodol 57:289–300.

Brämer GR. 1988. International statistical classification of diseases and related health problems. Tenth revision. World Health Stat Q Rapp Trimest Stat Sanit Mond 41:32–36.

Bult CJ, Eppig JT, Blake JA, Kadin JA, Richardson JE, Mouse Genome Database Group. 2016. Mouse genome database 2016. Nucleic Acids Res 44:D840-847.

Chatr-Aryamontri A, Breitkreutz B-J, Oughtred R, Boucher L, Heinicke S, Chen D, Stark C, Breitkreutz A, Kolas N, O'Donnell L, Reguly T, Nixon J, Ramage L, Winter A, Sellam A, Chang C, Hirschman J, Theesfeld C, Rust J, Livstone MS, Dolinski K, Tyers M. 2015. The BioGRID interaction database: 2015 update. Nucleic Acids Res 43:D470-478.

Chen W-H, Minguez P, Lercher MJ, Bork P. 2012. OGEE: an online gene essentiality database. Nucleic Acids Res 40:D901-906.

Fabregat A, Sidiropoulos K, Garapati P, Gillespie M, Hausmann K, Haw R, Jassal B, Jupe S, Korninger F, McKay S, Matthews L, May B, Milacic M, Rothfels K, Shamovsky V, Webber M, Weiser J, Williams M, Wu G, Stein L, Hermjakob H, D'Eustachio P. 2016. The Reactome pathway Knowledgebase. Nucleic Acids Res 44:D481-487.

Ge X, Kwok P-Y, Shieh JTC. 2015. Prioritizing genes for X-linked diseases using population exome data. Hum Mol Genet 24:599–608.

Gene Ontology Consortium. 2015. Gene Ontology Consortium: going forward. Nucleic Acids Res 43:D1049-1056.

Itan Y, Mazel M, Mazel B, Abhyankar A, Nitschke P, Quintana-Murci L, Boisson-Dupuis S, Boisson B, Abel L, Zhang S-Y, Casanova J-L. 2014. HGCS: an online tool for prioritizing disease-causing gene variants by biological distance. BMC Genomics 15:256.

Itan Y, Shang L, Boisson B, Ciancanelli MJ, Markle JG, Martinez-Barricarte R, Scott E, Shah I, Stenson PD, Gleeson J, Cooper DN, Quintana-Murci L, Zhang SY, Abel L, Casanova JL. 2016. The mutation significance cutoff: gene-level thresholds for variant predictions. Nat Methods 13:109–110.

Itan Y, Shang L, Boisson B, Patin E, Bolze A, Moncada-Vélez M, Scott E, Ciancanelli MJ, Lafaille FG, Markle JG, Martinez-Barricarte R, Jong

SJ de, Kong XF, Nitschke P, Belkadi A, Bustamante J, Puel A, Boisson-Dupuis S, Stenson PD, Gleeson JG, Cooper DN, Quintana-Murci L, Claverie JM, Zhang SY, Abel L, Casanova JL 2015. The human gene damage index as a gene-level approach to prioritizing exome variants. Proc Natl Acad Sci U S A 112:13615–13620.

Itan Y, Zhang S-Y, Vogt G, Abhyankar A, Herman M, Nitschke P, Fried D, Quintana-Murci L, Abel L, Casanova J-L. 2013. The human gene connectome as a map of short cuts for morbid allele discovery. Proc Natl Acad Sci U S A 110:5558–5563.

Jiao X, Sherman BT, Huang DW, Stephens R, Baseler MW, Lane HC, Lempicki RA. 2012. DAVID-WS: a stateful web service to facilitate gene/protein list analysis. Bioinforma Oxf Engl 28:1805–1806.

Khurana E, Fu Y, Chen J, Gerstein M. 2013. Interpretation of genomic variants using a unified biological network approach. PLoS Comput Biol 9:e1002886.

Liao B-Y, Zhang J. 2008. Null mutations in human and mouse orthologs frequently result in different phenotypes. Proc Natl Acad Sci U S A 105:6987–6992.

Petrovski S, Wang Q, Heinzen EL, Allen AS, Goldstein DB. 2013. Genic intolerance to functional variation and the interpretation of personal genomes. PLoS Genet 9:e1003709.

Samocha KE, Robinson EB, Sanders SJ, Stevens C, Sabo A, McGrath LM, Kosmicki JA, Rehnström K, Mallick S, Kirby A, Wall DP, MacArthur DG, Gabriel SB, DePristo M, Purcell SM, Palotie A, Boerwinkle E, Buxbaum JD, Cook EH Jr, Gibbs RA, Schellenberg GD, Sutcliffe JS, Devlin B, Roeder K, Neale BM, Daly MJ. 2014. A framework for the interpretation of de novo mutation in human disease. Nat Genet 46:944–950.
