# Supplementary material for: Properties of human genes guided by their enrichment in rare and common variants
Source: Hum Mutat. 2017 Dec 21;39(3):365–70. doi: 10.1002/humu.23377 (PMC5838408; doi:10.1002/humu.23377)
Supplement: Supplementary file 2 — Supplementary Table [file HUMU-39-365-s002.doc]

**Supplementary Table** Genes enriched in disease_causing variants (disease-EVset), rare variants (Rare-EVset) and common variants (Common-EVset) identified by the enrichment analysis. Gene names are from the HUGO Gene Nomenclature Committee (HGNC) Approved Gene Symbols.

| **Gene** | **Class of Enrichment** |
| --- | --- |
| ABCA3 | Rare-EVset |
| AOC2 | Rare-EVset |
| BSN | Rare-EVset |
| CHD7 | Rare-EVset |
| FLNB | Rare-EVset |
| ANKHD1 | Rare-EVset |
| CDC42BPB | Rare-EVset |
| CELSR3 | Rare-EVset |
| DOPEY2 | Rare-EVset |
| HECTD4 | Rare-EVset |
| IFT172 | Rare-EVset |
| IQCH | Rare-EVset |
| ITK | Rare-EVset |
| KMT2C | Rare-EVset |
| LAMB2 | Rare-EVset |
| LRBA | Rare-EVset |
| LRP1 | Rare-EVset |
| MAMDC4 | Rare-EVset |
| MYH2 | Rare-EVset |
| NOTCH1 | Rare-EVset |
| PITPNM1 | Rare-EVset |
| SGSM3 | Rare-EVset |
| SNCAIP | Rare-EVset |
| SPAG5 | Rare-EVset |
| SRCAP | Rare-EVset |
| SRRM2 | Rare-EVset |
| SYNE1 | Rare-EVset |
| TANC2 | Rare-EVset |
| TRRAP | Rare-EVset |
| USP54 | Rare-EVset |
| WDFY3 | Rare-EVset |
| WDR6 | Rare-EVset |
| AARS2 | Disease-EVset |
| ABCA1 | Disease-EVset |
| ABCA4 | Disease-EVset |
| ABCB4 | Disease-EVset |
| ABCB6 | Disease-EVset |
| ABCC6 | Disease-EVset |
| ABCC8 | Disease-EVset |
| ACADS | Disease-EVset |
| ACAT1 | Disease-EVset |
| ACP5 | Disease-EVset |
| ACTA1 | Disease-EVset |
| ACTA2 | Disease-EVset |
| ACTB | Disease-EVset |
| ACTC1 | Disease-EVset |
| ACTG1 | Disease-EVset |
| ACY1 | Disease-EVset |
| ADA | Disease-EVset |
| ADSL | Disease-EVset |
| AGA | Disease-EVset |
| AGXT | Disease-EVset |
| AICDA | Disease-EVset |
| AIRE | Disease-EVset |
| AK1 | Disease-EVset |
| ALAS2 | Disease-EVset |
| ALB | Disease-EVset |
| ALG1 | Disease-EVset |
| ALG6 | Disease-EVset |
| ALPL | Disease-EVset |
| ALX3 | Disease-EVset |
| ALX4 | Disease-EVset |
| AMH | Disease-EVset |
| AMN | Disease-EVset |
| AMT | Disease-EVset |
| ANG | Disease-EVset |
| ANKH | Disease-EVset |
| APC | Disease-EVset |
| APOE | Disease-EVset |
| APP | Disease-EVset |
| APRT | Disease-EVset |
| APTX | Disease-EVset |
| AQP2 | Disease-EVset |
| AR | Disease-EVset |
| ARSA | Disease-EVset |
| ARSB | Disease-EVset |
| ARSE | Disease-EVset |
| ARX | Disease-EVset |
| ASAH1 | Disease-EVset |
| ASL | Disease-EVset |
| ASPA | Disease-EVset |
| ASS1 | Disease-EVset |
| ASXL1 | Disease-EVset |
| ATL1 | Disease-EVset |
| ATM | Disease-EVset |
| ATP7A | Disease-EVset |
| ATP7B | Disease-EVset |
| ATR | Disease-EVset |
| ATRX | Disease-EVset |
| AVP | Disease-EVset |
| B2M | Disease-EVset |
| BBS1 | Disease-EVset |
| BBS10 | Disease-EVset |
| BBS2 | Disease-EVset |
| BBS4 | Disease-EVset |
| BBS5 | Disease-EVset |
| BCHE | Disease-EVset |
| BCS1L | Disease-EVset |
| BEST1 | Disease-EVset |
| BIN1 | Disease-EVset |
| BRAF | Disease-EVset |
| BRCA1 | Disease-EVset |
| BRCA2 | Disease-EVset |
| BRIP1 | Disease-EVset |
| BSND | Disease-EVset |
| BTK | Disease-EVset |
| CA2 | Disease-EVset |
| CAPN3 | Disease-EVset |
| CAPN5 | Disease-EVset |
| CASR | Disease-EVset |
| CAV3 | Disease-EVset |
| CBL | Disease-EVset |
| CBS | Disease-EVset |
| CCNO | Disease-EVset |
| CDH1 | Disease-EVset |
| CEBPA | Disease-EVset |
| CENPJ | Disease-EVset |
| CFB | Disease-EVset |
| CFH | Disease-EVset |
| CFTR | Disease-EVset |
| CHN1 | Disease-EVset |
| CLMP | Disease-EVset |
| CLN5 | Disease-EVset |
| CLN6 | Disease-EVset |
| COCH | Disease-EVset |
| COMP | Disease-EVset |
| COX7B | Disease-EVset |
| CP | Disease-EVset |
| CPOX | Disease-EVset |
| CPT1A | Disease-EVset |
| CPT2 | Disease-EVset |
| CRB1 | Disease-EVset |
| CRLF1 | Disease-EVset |
| CRX | Disease-EVset |
| CSRP3 | Disease-EVset |
| CSTB | Disease-EVset |
| CTC1 | Disease-EVset |
| CTNS | Disease-EVset |
| CTRC | Disease-EVset |
| CTSA | Disease-EVset |
| CTSC | Disease-EVset |
| CTSK | Disease-EVset |
| CUBN | Disease-EVset |
| CUL3 | Disease-EVset |
| CYBA | Disease-EVset |
| CYBB | Disease-EVset |
| DARS | Disease-EVset |
| DARS2 | Disease-EVset |
| DCX | Disease-EVset |
| DDC | Disease-EVset |
| DES | Disease-EVset |
| DKC1 | Disease-EVset |
| DLD | Disease-EVset |
| DOK7 | Disease-EVset |
| DOLK | Disease-EVset |
| DPYS | Disease-EVset |
| DSG2 | Disease-EVset |
| DUSP6 | Disease-EVset |
| EBP | Disease-EVset |
| EDA | Disease-EVset |
| EDAR | Disease-EVset |
| EDN1 | Disease-EVset |
| EFNB1 | Disease-EVset |
| EGR2 | Disease-EVset |
| ELAC2 | Disease-EVset |
| ELANE | Disease-EVset |
| EMD | Disease-EVset |
| EMG1 | Disease-EVset |
| ENG | Disease-EVset |
| ENPP1 | Disease-EVset |
| ERCC2 | Disease-EVset |
| ERF | Disease-EVset |
| ETHE1 | Disease-EVset |
| EXT1 | Disease-EVset |
| EYA1 | Disease-EVset |
| EZH2 | Disease-EVset |
| F10 | Disease-EVset |
| F11 | Disease-EVset |
| F12 | Disease-EVset |
| F2 | Disease-EVset |
| F7 | Disease-EVset |
| F8 | Disease-EVset |
| F9 | Disease-EVset |
| FAH | Disease-EVset |
| FAS | Disease-EVset |
| FBLN5 | Disease-EVset |
| FBN1 | Disease-EVset |
| FBN2 | Disease-EVset |
| FECH | Disease-EVset |
| FGD1 | Disease-EVset |
| FGF16 | Disease-EVset |
| FGF23 | Disease-EVset |
| FGF3 | Disease-EVset |
| FGF8 | Disease-EVset |
| FGFR1 | Disease-EVset |
| FGFR2 | Disease-EVset |
| FGFR3 | Disease-EVset |
| FH | Disease-EVset |
| FHL1 | Disease-EVset |
| FKRP | Disease-EVset |
| FLNA | Disease-EVset |
| FLRT3 | Disease-EVset |
| FOXC1 | Disease-EVset |
| FOXG1 | Disease-EVset |
| FOXL2 | Disease-EVset |
| FOXP3 | Disease-EVset |
| FSHR | Disease-EVset |
| FTL | Disease-EVset |
| FUCA1 | Disease-EVset |
| FUS | Disease-EVset |
| FXN | Disease-EVset |
| FZD4 | Disease-EVset |
| G6PC | Disease-EVset |
| G6PD | Disease-EVset |
| GAA | Disease-EVset |
| GALC | Disease-EVset |
| GALE | Disease-EVset |
| GALK1 | Disease-EVset |
| GALNS | Disease-EVset |
| GALT | Disease-EVset |
| GAN | Disease-EVset |
| GATM | Disease-EVset |
| GBA | Disease-EVset |
| GBE1 | Disease-EVset |
| GCDH | Disease-EVset |
| GCH1 | Disease-EVset |
| GCK | Disease-EVset |
| GDF1 | Disease-EVset |
| GDF2 | Disease-EVset |
| GDF3 | Disease-EVset |
| GDF5 | Disease-EVset |
| GDF6 | Disease-EVset |
| GDI1 | Disease-EVset |
| GFAP | Disease-EVset |
| GGCX | Disease-EVset |
| GH1 | Disease-EVset |
| GHR | Disease-EVset |
| GIPC3 | Disease-EVset |
| GJA1 | Disease-EVset |
| GJA3 | Disease-EVset |
| GJA5 | Disease-EVset |
| GJA8 | Disease-EVset |
| GJB1 | Disease-EVset |
| GJB2 | Disease-EVset |
| GJB3 | Disease-EVset |
| GJB4 | Disease-EVset |
| GJC2 | Disease-EVset |
| GLA | Disease-EVset |
| GLB1 | Disease-EVset |
| GLDC | Disease-EVset |
| GLRA1 | Disease-EVset |
| GNAS | Disease-EVset |
| GNE | Disease-EVset |
| GNS | Disease-EVset |
| GP1BA | Disease-EVset |
| GP9 | Disease-EVset |
| GPI | Disease-EVset |
| GRK1 | Disease-EVset |
| GSS | Disease-EVset |
| GUSB | Disease-EVset |
| HAMP | Disease-EVset |
| HBB | Disease-EVset |
| HCN1 | Disease-EVset |
| HEXA | Disease-EVset |
| HEXB | Disease-EVset |
| HFE | Disease-EVset |
| HFE2 | Disease-EVset |
| HGD | Disease-EVset |
| HINT1 | Disease-EVset |
| HLCS | Disease-EVset |
| HMBS | Disease-EVset |
| HRAS | Disease-EVset |
| HSF4 | Disease-EVset |
| HSPB1 | Disease-EVset |
| IDS | Disease-EVset |
| IDUA | Disease-EVset |
| IFIH1 | Disease-EVset |
| ABCB11 | Disease-EVset |
| ABCD1 | Disease-EVset |
| ABHD5 | Disease-EVset |
| ACAD8 | Disease-EVset |
| ACADM | Disease-EVset |
| ACADVL | Disease-EVset |
| ACVRL1 | Disease-EVset |
| ADAM10 | Disease-EVset |
| ADAMTS13 | Disease-EVset |
| AFG3L2 | Disease-EVset |
| AGPAT2 | Disease-EVset |
| ALDH3A2 | Disease-EVset |
| ALDH5A1 | Disease-EVset |
| ALDOB | Disease-EVset |
| ALOX12B | Disease-EVset |
| AMHR2 | Disease-EVset |
| AMPD3 | Disease-EVset |
| ANTXR2 | Disease-EVset |
| ATOH7 | Disease-EVset |
| ATP1A2 | Disease-EVset |
| ATP1A3 | Disease-EVset |
| ATP2A2 | Disease-EVset |
| ATP2C1 | Disease-EVset |
| ATP6V1B1 | Disease-EVset |
| ATP8B1 | Disease-EVset |
| AVPR2 | Disease-EVset |
| B3GALT6 | Disease-EVset |
| BHLHA9 | Disease-EVset |
| BMPR1A | Disease-EVset |
| BMPR2 | Disease-EVset |
| BUB1B | Disease-EVset |
| C19orf12 | Disease-EVset |
| CACNA1A | Disease-EVset |
| CACNA1F | Disease-EVset |
| CCND2 | Disease-EVset |
| CD40LG | Disease-EVset |
| CDAN1 | Disease-EVset |
| CDH23 | Disease-EVset |
| CDKN2A | Disease-EVset |
| CDKN3 | Disease-EVset |
| CEP290 | Disease-EVset |
| CHEK2 | Disease-EVset |
| CHRNA1 | Disease-EVset |
| CHRNE | Disease-EVset |
| CHST14 | Disease-EVset |
| CHST3 | Disease-EVset |
| CHST6 | Disease-EVset |
| CLCN1 | Disease-EVset |
| CLCN5 | Disease-EVset |
| CLCN7 | Disease-EVset |
| CLDN16 | Disease-EVset |
| CNGA3 | Disease-EVset |
| CNGB3 | Disease-EVset |
| CNNM4 | Disease-EVset |
| COL10A1 | Disease-EVset |
| COL1A1 | Disease-EVset |
| COL1A2 | Disease-EVset |
| COL2A1 | Disease-EVset |
| COL3A1 | Disease-EVset |
| COL4A3 | Disease-EVset |
| COL4A5 | Disease-EVset |
| COL7A1 | Disease-EVset |
| CRYGD | Disease-EVset |
| CTNNB1 | Disease-EVset |
| CYB5R3 | Disease-EVset |
| CYP11B2 | Disease-EVset |
| CYP17A1 | Disease-EVset |
| CYP1B1 | Disease-EVset |
| CYP21A2 | Disease-EVset |
| CYP27A1 | Disease-EVset |
| CYP27B1 | Disease-EVset |
| CYP2U1 | Disease-EVset |
| CYP4V2 | Disease-EVset |
| CYP7B1 | Disease-EVset |
| DHCR24 | Disease-EVset |
| DHCR7 | Disease-EVset |
| DHODH | Disease-EVset |
| DNMT3B | Disease-EVset |
| DPAGT1 | Disease-EVset |
| DYNC1H1 | Disease-EVset |
| EDNRB | Disease-EVset |
| EFEMP2 | Disease-EVset |
| EIF2B2 | Disease-EVset |
| EIF2B3 | Disease-EVset |
| EIF2B4 | Disease-EVset |
| EPM2A | Disease-EVset |
| FANCA | Disease-EVset |
| FKBP10 | Disease-EVset |
| FRMD7 | Disease-EVset |
| GATA1 | Disease-EVset |
| GATA6 | Disease-EVset |
| GHRHR | Disease-EVset |
| GLUD1 | Disease-EVset |
| GNA11 | Disease-EVset |
| GNPTAB | Disease-EVset |
| GNPTG | Disease-EVset |
| GNRHR | Disease-EVset |
| GPR143 | Disease-EVset |
| GPR179 | Disease-EVset |
| GRIN2B | Disease-EVset |
| GUCY2D | Disease-EVset |
| HADHB | Disease-EVset |
| HEPACAM | Disease-EVset |
| HGSNAT | Disease-EVset |
| HMGCL | Disease-EVset |
| HNF1A | Disease-EVset |
| HNF1B | Disease-EVset |
| HOXD13 | Disease-EVset |
| HPRT1 | Disease-EVset |
| HSD11B2 | Disease-EVset |
| HSD17B10 | Disease-EVset |
| HSD17B3 | Disease-EVset |
| HSD3B2 | Disease-EVset |
| HTRA1 | Disease-EVset |
| IER3IP1 | Disease-EVset |
| IFNGR1 | Disease-EVset |
| IGHMBP2 | Disease-EVset |
| IHH | Disease-EVset |
| IKBKG | Disease-EVset |
| IL2RG | Disease-EVset |
| IMPAD1 | Disease-EVset |
| INS | Disease-EVset |
| INSR | Disease-EVset |
| IRF6 | Disease-EVset |
| ITGA2B | Disease-EVset |
| ITGB2 | Disease-EVset |
| ITGB3 | Disease-EVset |
| ITM2B | Disease-EVset |
| IVD | Disease-EVset |
| JAG1 | Disease-EVset |
| JAGN1 | Disease-EVset |
| JPH2 | Disease-EVset |
| KCNA1 | Disease-EVset |
| KCNE1 | Disease-EVset |
| KCNE2 | Disease-EVset |
| KCNH2 | Disease-EVset |
| KCNJ1 | Disease-EVset |
| KCNJ10 | Disease-EVset |
| KCNJ11 | Disease-EVset |
| KCNJ13 | Disease-EVset |
| KCNJ18 | Disease-EVset |
| KCNJ2 | Disease-EVset |
| KCNK3 | Disease-EVset |
| KCNQ1 | Disease-EVset |
| KCNQ2 | Disease-EVset |
| KCTD7 | Disease-EVset |
| KDM5C | Disease-EVset |
| KERA | Disease-EVset |
| KIF11 | Disease-EVset |
| KIF5A | Disease-EVset |
| KISS1R | Disease-EVset |
| KIT | Disease-EVset |
| KLF1 | Disease-EVset |
| KLHL3 | Disease-EVset |
| KRAS | Disease-EVset |
| KRT1 | Disease-EVset |
| KRT10 | Disease-EVset |
| KRT12 | Disease-EVset |
| KRT14 | Disease-EVset |
| KRT16 | Disease-EVset |
| KRT17 | Disease-EVset |
| KRT2 | Disease-EVset |
| KRT5 | Disease-EVset |
| KRT6A | Disease-EVset |
| KRT9 | Disease-EVset |
| L1CAM | Disease-EVset |
| L2HGDH | Disease-EVset |
| LCAT | Disease-EVset |
| LDLR | Disease-EVset |
| LGI1 | Disease-EVset |
| LHCGR | Disease-EVset |
| LHX3 | Disease-EVset |
| LITAF | Disease-EVset |
| LMNA | Disease-EVset |
| LMX1B | Disease-EVset |
| LPAR6 | Disease-EVset |
| LPL | Disease-EVset |
| LRP5 | Disease-EVset |
| LRRC6 | Disease-EVset |
| LRRK2 | Disease-EVset |
| LTBP2 | Disease-EVset |
| LYZ | Disease-EVset |
| MAB21L2 | Disease-EVset |
| MAFB | Disease-EVset |
| MAK | Disease-EVset |
| MAN2B1 | Disease-EVset |
| MAP2K1 | Disease-EVset |
| MAPT | Disease-EVset |
| MAT1A | Disease-EVset |
| MATN3 | Disease-EVset |
| MBTPS2 | Disease-EVset |
| MC2R | Disease-EVset |
| MC4R | Disease-EVset |
| MCCC2 | Disease-EVset |
| MCOLN1 | Disease-EVset |
| MECP2 | Disease-EVset |
| MEFV | Disease-EVset |
| MEN1 | Disease-EVset |
| MESP2 | Disease-EVset |
| MET | Disease-EVset |
| MFN2 | Disease-EVset |
| MKKS | Disease-EVset |
| MLC1 | Disease-EVset |
| MLH1 | Disease-EVset |
| MMAA | Disease-EVset |
| MMACHC | Disease-EVset |
| MMADHC | Disease-EVset |
| MOCS2 | Disease-EVset |
| MPI | Disease-EVset |
| MPLKIP | Disease-EVset |
| MPZ | Disease-EVset |
| MSH2 | Disease-EVset |
| MSX1 | Disease-EVset |
| MT-ATP6 | Disease-EVset |
| MT-CO1 | Disease-EVset |
| MT-CO3 | Disease-EVset |
| MT-CYB | Disease-EVset |
| MT-ND1 | Disease-EVset |
| MT-ND2 | Disease-EVset |
| MT-ND3 | Disease-EVset |
| MT-ND4 | Disease-EVset |
| MT-ND5 | Disease-EVset |
| MT-ND6 | Disease-EVset |
| MTHFR | Disease-EVset |
| MTM1 | Disease-EVset |
| MUT | Disease-EVset |
| MUTYH | Disease-EVset |
| MVK | Disease-EVset |
| MYBPC3 | Disease-EVset |
| MYH3 | Disease-EVset |
| MYH7 | Disease-EVset |
| MYH9 | Disease-EVset |
| MYL2 | Disease-EVset |
| MYL3 | Disease-EVset |
| MYO7A | Disease-EVset |
| MYOC | Disease-EVset |
| NAGLU | Disease-EVset |
| NAGS | Disease-EVset |
| NANOS1 | Disease-EVset |
| NCF1 | Disease-EVset |
| NDP | Disease-EVset |
| NDUFA1 | Disease-EVset |
| NDUFS1 | Disease-EVset |
| NEFL | Disease-EVset |
| NEU1 | Disease-EVset |
| NF1 | Disease-EVset |
| NF2 | Disease-EVset |
| NHLRC1 | Disease-EVset |
| NIPBL | Disease-EVset |
| NKX2-5 | Disease-EVset |
| NOBOX | Disease-EVset |
| NOG | Disease-EVset |
| NOTCH3 | Disease-EVset |
| NPC1 | Disease-EVset |
| NPC2 | Disease-EVset |
| NPHP4 | Disease-EVset |
| NPHS1 | Disease-EVset |
| NPR2 | Disease-EVset |
| NR0B1 | Disease-EVset |
| NR2E3 | Disease-EVset |
| NR2F1 | Disease-EVset |
| NR2F2 | Disease-EVset |
| NR3C1 | Disease-EVset |
| NR3C2 | Disease-EVset |
| NRAS | Disease-EVset |
| NSD1 | Disease-EVset |
| NYX | Disease-EVset |
| OAT | Disease-EVset |
| OCA2 | Disease-EVset |
| OCRL | Disease-EVset |
| OFD1 | Disease-EVset |
| OPA1 | Disease-EVset |
| OPN1LW | Disease-EVset |
| ORAI1 | Disease-EVset |
| OTC | Disease-EVset |
| OTX2 | Disease-EVset |
| OXCT1 | Disease-EVset |
| PAFAH1B1 | Disease-EVset |
| PAH | Disease-EVset |
| PALB2 | Disease-EVset |
| PANK2 | Disease-EVset |
| PARK2 | Disease-EVset |
| PARK7 | Disease-EVset |
| PAX3 | Disease-EVset |
| PAX6 | Disease-EVset |
| PAX9 | Disease-EVset |
| PCCA | Disease-EVset |
| PCCB | Disease-EVset |
| PCDH19 | Disease-EVset |
| PCYT1A | Disease-EVset |
| PDHA1 | Disease-EVset |
| PDX1 | Disease-EVset |
| PEX10 | Disease-EVset |
| PEX5 | Disease-EVset |
| PEX7 | Disease-EVset |
| PFN1 | Disease-EVset |
| PGAM2 | Disease-EVset |
| PGK1 | Disease-EVset |
| PHC1 | Disease-EVset |
| PHEX | Disease-EVset |
| PHF6 | Disease-EVset |
| PHGDH | Disease-EVset |
| PHKA2 | Disease-EVset |
| PHYH | Disease-EVset |
| PIGA | Disease-EVset |
| PIGL | Disease-EVset |
| PIK3CA | Disease-EVset |
| PINK1 | Disease-EVset |
| PITX2 | Disease-EVset |
| PKD1 | Disease-EVset |
| PKD2 | Disease-EVset |
| PKHD1 | Disease-EVset |
| PKLR | Disease-EVset |
| PLG | Disease-EVset |
| PLOD1 | Disease-EVset |
| PLP1 | Disease-EVset |
| PMM2 | Disease-EVset |
| PMP22 | Disease-EVset |
| PMS2 | Disease-EVset |
| PNKP | Disease-EVset |
| PNP | Disease-EVset |
| POLG | Disease-EVset |
| POLH | Disease-EVset |
| POLR3A | Disease-EVset |
| POLR3B | Disease-EVset |
| POMGNT1 | Disease-EVset |
| POMT1 | Disease-EVset |
| POMT2 | Disease-EVset |
| POR | Disease-EVset |
| PORCN | Disease-EVset |
| POU1F1 | Disease-EVset |
| POU3F4 | Disease-EVset |
| PPT1 | Disease-EVset |
| PRCD | Disease-EVset |
| PRKAG2 | Disease-EVset |
| PRKAR1A | Disease-EVset |
| PRKCG | Disease-EVset |
| PRNP | Disease-EVset |
| PROC | Disease-EVset |
| PROKR2 | Disease-EVset |
| PROP1 | Disease-EVset |
| PROS1 | Disease-EVset |
| PRPF3 | Disease-EVset |
| PRPF6 | Disease-EVset |
| PRPH2 | Disease-EVset |
| PRPS1 | Disease-EVset |
| PRSS1 | Disease-EVset |
| PRSS56 | Disease-EVset |
| PSEN1 | Disease-EVset |
| PTCH1 | Disease-EVset |
| PTEN | Disease-EVset |
| PTPN11 | Disease-EVset |
| PTPRT | Disease-EVset |
| PTS | Disease-EVset |
| PURA | Disease-EVset |
| PYCR1 | Disease-EVset |
| PYGM | Disease-EVset |
| QDPR | Disease-EVset |
| RAB39B | Disease-EVset |
| RAD21 | Disease-EVset |
| RAF1 | Disease-EVset |
| RAG1 | Disease-EVset |
| RAPSN | Disease-EVset |
| RB1 | Disease-EVset |
| RBM8A | Disease-EVset |
| RBP3 | Disease-EVset |
| RBP4 | Disease-EVset |
| RDH12 | Disease-EVset |
| RDH5 | Disease-EVset |
| RECQL4 | Disease-EVset |
| REN | Disease-EVset |
| RET | Disease-EVset |
| RHO | Disease-EVset |
| RNASEH2A | Disease-EVset |
| RNASEH2B | Disease-EVset |
| ROR2 | Disease-EVset |
| RP2 | Disease-EVset |
| RPE65 | Disease-EVset |
| RPGR | Disease-EVset |
| RPL11 | Disease-EVset |
| RPS17 | Disease-EVset |
| RPS19 | Disease-EVset |
| RPS6KA3 | Disease-EVset |
| RRM2B | Disease-EVset |
| RS1 | Disease-EVset |
| RTEL1 | Disease-EVset |
| RUNX2 | Disease-EVset |
| RYR1 | Disease-EVset |
| RYR2 | Disease-EVset |
| SACS | Disease-EVset |
| SAR1B | Disease-EVset |
| SBDS | Disease-EVset |
| SCARF2 | Disease-EVset |
| SCN1A | Disease-EVset |
| SCN4A | Disease-EVset |
| SCN5A | Disease-EVset |
| SCNN1B | Disease-EVset |
| SDHA | Disease-EVset |
| SDHAF1 | Disease-EVset |
| SDHB | Disease-EVset |
| SDHD | Disease-EVset |
| SERPINA7 | Disease-EVset |
| SERPINC1 | Disease-EVset |
| SERPING1 | Disease-EVset |
| SFTPC | Disease-EVset |
| SGCA | Disease-EVset |
| SGCB | Disease-EVset |
| SGSH | Disease-EVset |
| SH2D1A | Disease-EVset |
| SHANK3 | Disease-EVset |
| SHH | Disease-EVset |
| SHOX | Disease-EVset |
| SIX1 | Disease-EVset |
| SIX3 | Disease-EVset |
| SLC12A3 | Disease-EVset |
| SLC16A2 | Disease-EVset |
| SLC17A5 | Disease-EVset |
| SLC19A2 | Disease-EVset |
| SLC22A12 | Disease-EVset |
| SLC22A5 | Disease-EVset |
| SLC25A1 | Disease-EVset |
| SLC25A15 | Disease-EVset |
| SLC25A20 | Disease-EVset |
| SLC25A4 | Disease-EVset |
| SLC26A2 | Disease-EVset |
| SLC26A4 | Disease-EVset |
| SLC2A1 | Disease-EVset |
| SLC2A10 | Disease-EVset |
| SLC30A10 | Disease-EVset |
| SLC35A2 | Disease-EVset |
| SLC37A4 | Disease-EVset |
| SLC39A4 | Disease-EVset |
| SLC3A1 | Disease-EVset |
| SLC40A1 | Disease-EVset |
| SLC45A2 | Disease-EVset |
| SLC46A1 | Disease-EVset |
| SLC4A1 | Disease-EVset |
| SLC4A11 | Disease-EVset |
| SLC5A5 | Disease-EVset |
| SLC6A5 | Disease-EVset |
| SLC6A8 | Disease-EVset |
| SLC7A14 | Disease-EVset |
| SLC7A7 | Disease-EVset |
| SLC7A9 | Disease-EVset |
| SLITRK6 | Disease-EVset |
| SLURP1 | Disease-EVset |
| SMAD3 | Disease-EVset |
| SMAD4 | Disease-EVset |
| SMARCA2 | Disease-EVset |
| SMARCAL1 | Disease-EVset |
| SMC1A | Disease-EVset |
| SMC3 | Disease-EVset |
| SMN1 | Disease-EVset |
| SMPD1 | Disease-EVset |
| SMPX | Disease-EVset |
| SNRNP200 | Disease-EVset |
| SNRPE | Disease-EVset |
| SOD1 | Disease-EVset |
| SOS1 | Disease-EVset |
| SOST | Disease-EVset |
| SOX18 | Disease-EVset |
| SOX9 | Disease-EVset |
| SPAST | Disease-EVset |
| SPINK1 | Disease-EVset |
| SPR | Disease-EVset |
| SPRED1 | Disease-EVset |
| SPTA1 | Disease-EVset |
| SRD5A2 | Disease-EVset |
| SRD5A3 | Disease-EVset |
| SRY | Disease-EVset |
| STAR | Disease-EVset |
| STAT3 | Disease-EVset |
| STK11 | Disease-EVset |
| STS | Disease-EVset |
| STX1B | Disease-EVset |
| SUMF1 | Disease-EVset |
| SUOX | Disease-EVset |
| SURF1 | Disease-EVset |
| SYNGAP1 | Disease-EVset |
| SYP | Disease-EVset |
| TACSTD2 | Disease-EVset |
| TARDBP | Disease-EVset |
| TAZ | Disease-EVset |
| TBX22 | Disease-EVset |
| TCAP | Disease-EVset |
| TCF4 | Disease-EVset |
| TERT | Disease-EVset |
| TFAP2B | Disease-EVset |
| TGDS | Disease-EVset |
| TGFB1 | Disease-EVset |
| TGFBI | Disease-EVset |
| TGFBR1 | Disease-EVset |
| TGFBR2 | Disease-EVset |
| TGM1 | Disease-EVset |
| THBD | Disease-EVset |
| THRB | Disease-EVset |
| TIMM8A | Disease-EVset |
| TIMP3 | Disease-EVset |
| TK2 | Disease-EVset |
| TMC1 | Disease-EVset |
| TMEM240 | Disease-EVset |
| TMIE | Disease-EVset |
| TNFRSF1A | Disease-EVset |
| TNNI3 | Disease-EVset |
| TNNT2 | Disease-EVset |
| TP53 | Disease-EVset |
| TP63 | Disease-EVset |
| TPI1 | Disease-EVset |
| TPM1 | Disease-EVset |
| TPM2 | Disease-EVset |
| TPMT | Disease-EVset |
| TPO | Disease-EVset |
| TPP1 | Disease-EVset |
| TRAPPC2 | Disease-EVset |
| TREM2 | Disease-EVset |
| TREX1 | Disease-EVset |
| TRNT1 | Disease-EVset |
| TSC1 | Disease-EVset |
| TSC2 | Disease-EVset |
| TSHR | Disease-EVset |
| TTPA | Disease-EVset |
| TTR | Disease-EVset |
| TUBA1A | Disease-EVset |
| TUBB | Disease-EVset |
| TUBB2B | Disease-EVset |
| TULP1 | Disease-EVset |
| TWIST1 | Disease-EVset |
| TYMP | Disease-EVset |
| TYR | Disease-EVset |
| UBIAD1 | Disease-EVset |
| UBQLN2 | Disease-EVset |
| UGT1A1 | Disease-EVset |
| UMOD | Disease-EVset |
| UROD | Disease-EVset |
| UROS | Disease-EVset |
| VCP | Disease-EVset |
| VDR | Disease-EVset |
| VEGFC | Disease-EVset |
| VHL | Disease-EVset |
| VWF | Disease-EVset |
| WAS | Disease-EVset |
| WDR19 | Disease-EVset |
| WDR34 | Disease-EVset |
| WFS1 | Disease-EVset |
| WISP3 | Disease-EVset |
| WNT1 | Disease-EVset |
| WNT10A | Disease-EVset |
| WT1 | Disease-EVset |
| XK | Disease-EVset |
| XPA | Disease-EVset |
| ZFP57 | Disease-EVset |
| ZFPM2 | Disease-EVset |
| ZIC2 | Disease-EVset |
| ZIC3 | Disease-EVset |
| ZMYND10 | Disease-EVset |
| ABO | Common-EVset |
| ACAN | Common-EVset |
| ACP6 | Common-EVset |
| ALPK2 | Common-EVset |
| APOL4 | Common-EVset |
| ARL5C | Common-EVset |
| BST1 | Common-EVset |
| BTNL2 | Common-EVset |
| CA6 | Common-EVset |
| CD177 | Common-EVset |
| CD207 | Common-EVset |
| CD6 | Common-EVset |
| CDSN | Common-EVset |
| CEP55 | Common-EVset |
| CHIA | Common-EVset |
| CPA5 | Common-EVset |
| CTSE | Common-EVset |
| DCHS2 | Common-EVset |
| DPCR1 | Common-EVset |
| DSPP | Common-EVset |
| FLG | Common-EVset |
| FMO2 | Common-EVset |
| FRAS1 | Common-EVset |
| FSIP2 | Common-EVset |
| GCSH | Common-EVset |
| H3F3C | Common-EVset |
| HAP1 | Common-EVset |
| HCFC1 | Common-EVset |
| HCN2 | Common-EVset |
| HLA-A | Common-EVset |
| HLA-B | Common-EVset |
| HLA-C | Common-EVset |
| HRNR | Common-EVset |
| IBSP | Common-EVset |
| ACSBG2 | Common-EVset |
| AHNAK2 | Common-EVset |
| AKR1C3 | Common-EVset |
| AMACR | Common-EVset |
| ANKDD1B | Common-EVset |
| ANKRD31 | Common-EVset |
| ANKRD33B | Common-EVset |
| ANKRD35 | Common-EVset |
| ANKRD36 | Common-EVset |
| ANKRD36B | Common-EVset |
| ANKRD53 | Common-EVset |
| AQP12B | Common-EVset |
| ARMCX4 | Common-EVset |
| AVPR1B | Common-EVset |
| BTN3A2 | Common-EVset |
| C10orf71 | Common-EVset |
| C1orf167 | Common-EVset |
| C6orf10 | Common-EVset |
| C6orf15 | Common-EVset |
| C9orf66 | Common-EVset |
| CCDC137 | Common-EVset |
| CCDC154 | Common-EVset |
| CCDC177 | Common-EVset |
| CCHCR1 | Common-EVset |
| CEACAM20 | Common-EVset |
| CEACAM3 | Common-EVset |
| CENPU | Common-EVset |
| CEP295 | Common-EVset |
| CHAMP1 | Common-EVset |
| CHD1L | Common-EVset |
| CHST13 | Common-EVset |
| CITED4 | Common-EVset |
| CLCNKB | Common-EVset |
| CLDN24 | Common-EVset |
| COL18A1 | Common-EVset |
| CYP4F12 | Common-EVset |
| DHRS4L2 | Common-EVset |
| DNAH12 | Common-EVset |
| DNAH14 | Common-EVset |
| FAM153A | Common-EVset |
| FAM181B | Common-EVset |
| FAM184B | Common-EVset |
| FAM186A | Common-EVset |
| FAM188B | Common-EVset |
| FAM189A1 | Common-EVset |
| FAM205A | Common-EVset |
| FAM25A | Common-EVset |
| FAM86B1 | Common-EVset |
| FAM86C1 | Common-EVset |
| FBRSL1 | Common-EVset |
| GOLGA6L2 | Common-EVset |
| GPRIN2 | Common-EVset |
| GPSM1 | Common-EVset |
| GRIN3B | Common-EVset |
| GUCA1C | Common-EVset |
| HLA-DOB | Common-EVset |
| HLA-DPA1 | Common-EVset |
| HLA-DPB1 | Common-EVset |
| HLA-DQA1 | Common-EVset |
| HLA-DQB1 | Common-EVset |
| HLA-DRB1 | Common-EVset |
| HLA-DRB5 | Common-EVset |
| HMCN2 | Common-EVset |
| HSPA1A | Common-EVset |
| IGFN1 | Common-EVset |
| IQSEC3 | Common-EVset |
| IRAK1 | Common-EVset |
| KANK3 | Common-EVset |
| KIAA0753 | Common-EVset |
| KIAA1456 | Common-EVset |
| KIR3DL1 | Common-EVset |
| KLHDC7A | Common-EVset |
| KLRC3 | Common-EVset |
| KRT40 | Common-EVset |
| KRTAP1-1 | Common-EVset |
| KRTAP10-1 | Common-EVset |
| KRTAP10-10 | Common-EVset |
| KRTAP10-5 | Common-EVset |
| KRTAP10-9 | Common-EVset |
| KRTAP12-2 | Common-EVset |
| KRTAP4-11 | Common-EVset |
| KRTAP4-4 | Common-EVset |
| KRTAP4-5 | Common-EVset |
| KRTAP4-8 | Common-EVset |
| KRTAP5-5 | Common-EVset |
| KRTAP9-6 | Common-EVset |
| LEFTY1 | Common-EVset |
| LILRB1 | Common-EVset |
| LILRB3 | Common-EVset |
| LRRC53 | Common-EVset |
| LY6G5B | Common-EVset |
| LY75 | Common-EVset |
| MAGEB16 | Common-EVset |
| MAGEC1 | Common-EVset |
| MALRD1 | Common-EVset |
| MAPK15 | Common-EVset |
| MCCD1 | Common-EVset |
| MDC1 | Common-EVset |
| MESP1 | Common-EVset |
| METTL4 | Common-EVset |
| MGAM | Common-EVset |
| MICA | Common-EVset |
| MICB | Common-EVset |
| MKI67 | Common-EVset |
| MLXIPL | Common-EVset |
| MROH2A | Common-EVset |
| MUC12 | Common-EVset |
| MUC16 | Common-EVset |
| MUC20 | Common-EVset |
| MUC21 | Common-EVset |
| MUC22 | Common-EVset |
| MUC4 | Common-EVset |
| MUC5B | Common-EVset |
| NACAD | Common-EVset |
| NBPF3 | Common-EVset |
| NOTCH4 | Common-EVset |
| NT5C3B | Common-EVset |
| NUTM2A | Common-EVset |
| NUTM2F | Common-EVset |
| OR10C1 | Common-EVset |
| OR10G7 | Common-EVset |
| OR12D2 | Common-EVset |
| OR13C5 | Common-EVset |
| OR14A16 | Common-EVset |
| OR1I1 | Common-EVset |
| OR1L1 | Common-EVset |
| OR1L6 | Common-EVset |
| OR1N2 | Common-EVset |
| OR2D3 | Common-EVset |
| OR2L3 | Common-EVset |
| OR2L8 | Common-EVset |
| OR2T12 | Common-EVset |
| OR2T27 | Common-EVset |
| OR2T29 | Common-EVset |
| OR2T4 | Common-EVset |
| OR2T5 | Common-EVset |
| OR2T8 | Common-EVset |
| OR51A2 | Common-EVset |
| OR51B6 | Common-EVset |
| OR51F1 | Common-EVset |
| OR51M1 | Common-EVset |
| OR52L1 | Common-EVset |
| OR5B3 | Common-EVset |
| OR5H14 | Common-EVset |
| OR5H6 | Common-EVset |
| OR5K3 | Common-EVset |
| OR5P2 | Common-EVset |
| OR5W2 | Common-EVset |
| OR6K6 | Common-EVset |
| OR7A10 | Common-EVset |
| OR8D4 | Common-EVset |
| OR8H3 | Common-EVset |
| OTOG | Common-EVset |
| OVCH2 | Common-EVset |
| PARP10 | Common-EVset |
| PARP4 | Common-EVset |
| PCDHA10 | Common-EVset |
| PCDHA13 | Common-EVset |
| PCDHA3 | Common-EVset |
| PCDHA4 | Common-EVset |
| PCDHA9 | Common-EVset |
| PCDHB3 | Common-EVset |
| PCDHB6 | Common-EVset |
| PCDHB7 | Common-EVset |
| PCDHB8 | Common-EVset |
| PCDHB9 | Common-EVset |
| PDE4DIP | Common-EVset |
| PERM1 | Common-EVset |
| PIEZO1 | Common-EVset |
| PKD1L3 | Common-EVset |
| PLEC | Common-EVset |
| PLIN4 | Common-EVset |
| PLK5 | Common-EVset |
| PNLIPRP1 | Common-EVset |
| PNLIPRP3 | Common-EVset |
| POM121L2 | Common-EVset |
| POTED | Common-EVset |
| POTEI | Common-EVset |
| POU5F1 | Common-EVset |
| POU5F1B | Common-EVset |
| PRAMEF10 | Common-EVset |
| PRAMEF2 | Common-EVset |
| PRAMEF4 | Common-EVset |
| PRR21 | Common-EVset |
| PRR25 | Common-EVset |
| PSORS1C1 | Common-EVset |
| PTCHD3 | Common-EVset |
| PTX4 | Common-EVset |
| RBMXL3 | Common-EVset |
| RFPL2 | Common-EVset |
| RFPL3 | Common-EVset |
| RNF39 | Common-EVset |
| RTP4 | Common-EVset |
| SDR39U1 | Common-EVset |
| SERPINB11 | Common-EVset |
| SIGLEC12 | Common-EVset |
| SIRPA | Common-EVset |
| SIRPB1 | Common-EVset |
| SLC22A31 | Common-EVset |
| SLITRK2 | Common-EVset |
| SOWAHD | Common-EVset |
| SPINK5 | Common-EVset |
| SSC5D | Common-EVset |
| SSPO | Common-EVset |
| STARD9 | Common-EVset |
| STEAP1B | Common-EVset |
| SYCE1L | Common-EVset |
| SYNM | Common-EVset |
| TAP1 | Common-EVset |
| TAP2 | Common-EVset |
| TAS2R31 | Common-EVset |
| TAS2R42 | Common-EVset |
| TCP10 | Common-EVset |
| THEGL | Common-EVset |
| TLR6 | Common-EVset |
| TNFRSF10D | Common-EVset |
| TPPP | Common-EVset |
| TPSD1 | Common-EVset |
| TRIM31 | Common-EVset |
| TRIM40 | Common-EVset |
| TRIM64C | Common-EVset |
| TSEN54 | Common-EVset |
| TSGA10IP | Common-EVset |
| TSPAN10 | Common-EVset |
| TSPEAR | Common-EVset |
| UBD | Common-EVset |
| UGT1A5 | Common-EVset |
| USP17L7 | Common-EVset |
| USP17L8 | Common-EVset |
| USP41 | Common-EVset |
| VARS2 | Common-EVset |
| VCX2 | Common-EVset |
| VWDE | Common-EVset |
| WBSCR27 | Common-EVset |
| WBSCR28 | Common-EVset |
| ZC3H12D | Common-EVset |
| ZNF468 | Common-EVset |
| ZNF469 | Common-EVset |
| ZNF510 | Common-EVset |
| ZNF518A | Common-EVset |
| ZNF568 | Common-EVset |
| ZNF577 | Common-EVset |
| ZNF626 | Common-EVset |
| ZNF718 | Common-EVset |
| ZNF737 | Common-EVset |
| ZNF844 | Common-EVset |
| ZNF853 | Common-EVset |
| ZNF90 | Common-EVset |
